# Safe, effective and cost-effective oxygen saturation targets for children and adolescents with respiratory distress: protocol for a randomised controlled trial (OxyKids study)

**DOI:** 10.1136/bmjopen-2024-087891

**Published:** 2024-12-22

**Authors:** Sam Louman, Karlijn J van Stralen, Gerard Koppelman, Anja Vaessen-Verberne, Jolita Bekhof, J Bosmans, Caroline Brackel, Arvid W A Kamps, Ismé de Kleer, Walter Balemans, Mirjam Scheffer, Marianne Brouwer, Marije van den Beukel, Eleni-Rosalina Andrinopoulou, Mariëlle W H Pijnenburg, Annemie L M Boehmer

**Affiliations:** 1 Spaarne Gasthuis Academy, Hoofddorp, The Netherlands; 2 Department of Paediatrics/Division of Paediatric Respiratory Medicine and Allergology, Erasmus MC Sophia Children Hospital, Rotterdam, The Netherlands; 3 Department of Paediatric Pulmonolgy and Paediatric Allergology, University Medical Centre, Groningen, The Netherlands; 4 Groningen Research Institute for Asthma and COPD, University of Groningen, Groningen, The Netherlands; 5 Department of Paediatrics, Amphia Hospital, Breda, The Netherlands; 6 Department of Paediatrics, Isala Hospital, Zwolle, The Netherlands; 7 Department of Health Sciences, Amsterdam Public Health Research Institute, Vrije Universiteit Amsterdam, Amsterdam, The Netherlands; 8 Department of Paediatrics, Tergooi Medical Centre, Hilversum, The Netherlands; 9 Department of Paediatrics, Martini Hospital, Groningen, The Netherlands; 10 Department of Paediatrics, Franciscus Gasthuis, Rotterdam, The Netherlands; 11 Department of Paediatrics, St Antonius Hospital Nieuwegein, Nieuwegein, The Netherlands; 12 Department of Paediatrics, Rijnstate Hospital, Arnhem, The Netherlands; 13 Pediatrics, Canisius Wilhelmina Ziekenhuis, Nijmegen, The Netherlands; 14 Department of Paediatrics, Haaglanden Medical Center Bronovo, Den Haag, The Netherlands; 15 Department of Biostatistics, Department of Epidemiology, Erasmus Medical Center, Rotterdam, The Netherlands; 16 Department of Paediatrics, Spaarne Gasthuis, Haarlem, The Netherlands

**Keywords:** PAEDIATRICS, Child, Oxygen Saturation, Clinical Trial, RESPIRATORY MEDICINE (see Thoracic Medicine)

## Abstract

**Introduction:**

Little is known about the effectiveness and safety of oxygen saturation (SpO2) thresholds in children admitted with respiratory distress. The current 90%–94% threshold could lead to prolonged administration of supplemental oxygen, increased duration of hospital admissions, distress for children and families, and healthcare costs. To balance reducing unnecessary oxygen administration and preventing hypoxia, a lower SpO2 threshold of 88% for oxygen supplementation in children has been suggested. This trial aims to test the hypothesis that a lower SpO2 threshold of 88% safely reduces the length of hospital stay in children admitted with respiratory distress when compared with a 92% SpO2 threshold and to assess its cost-effectiveness.

**Methods and analysis:**

This is a multicentre, open-label, randomised controlled trial with two parallel arms. A total of 560 children aged 6 weeks to 12 years admitted with a diagnosis of bronchiolitis, viral wheeze or lower respiratory tract infection will be recruited and equally randomised into an intervention or usual care arm. Intervention arm patients will receive supplemental oxygen if SpO2 falls below 88% or above 88% if deemed necessary by clinical staff. Control arm patients will receive supplemental oxygen if SpO2 falls below 92% or above 92% if deemed necessary by staff. The primary outcome is the time from admission to the time when all prespecified discharge criteria are met. Secondary outcomes are length of stay, safety (time to recovery, readmissions and paediatric intensive care admissions), quality of life, parental anxiety and societal costs. Patients are followed up by digital questionnaires up to 90 days postdischarge.

**Ethics and dissemination:**

This study has received approval from the research ethics committee (REC) of Leiden, Den Haag and Delft (EU CT number 2023-504817-56). Written informed consent will be obtained from parents or guardians. Parents of patients and patient representatives are involved in all stages of the study, from design to results, interpretation and dissemination. The results of this trial will be disseminated via lay publications, peer-reviewed scientific journals and academic conferences.

**Trial registration number:**

NCT06016244.

**STRENGTHS AND LIMITATIONS OF THIS STUDY**

- This pragmatic trial and study procedures are very close to real-life practice, improving the generalisation of results and future implementation.
- The results of this study will enable the implementation of uniform oxygen saturation (SpO2) thresholds for nearly all children with respiratory distress due to a respiratory tract infection or viral wheezing, thanks to the broad age group and various diagnoses in the study population.
- Asthma patients aged 6 years and older are not included as there are safety concerns about lower SpO2 thresholds during the acute exacerbation phase.
- The open-label design introduces potential biases, which we will minimise by registering a fixed set of clinical parameters at oxygen start and stop to quantify potential biases, as well as a thorough training of physicians, nurses and trial staff.
- Safety evaluations in this study do not cover the long-term neurocognitive effects associated with either a lower or higher saturation threshold, as only short-term follow-up is described. However, follow-up studies are warranted to address the potential neurocognitive effects of lower or higher saturation thresholds in the long term.

## Introduction

 Acute respiratory distress is a common reason for hospital admission in children, caused by a variety of underlying diseases like bronchiolitis, viral wheeze, asthma or pneumonia.^1^ Primary treatment is the administration of supplemental oxygen.

Safe and effective oxygen therapy in children is crucial, as both undertreatment and overtreatment can have serious consequences. Supplemental oxygen is key in preventing tissue hypoxia during respiratory distress. However, overtreatment can lead to hyperoxia and oxidative stress, as shown in both mechanically ventilated children and adults.^2 3^ Moreover, aiming at higher oxygen saturations (SpO2) can prolong hospitalisation and cause unnecessary discomfort.

In clinical practice, supplemental oxygen is titrated on both respiratory distress and the recommended SpO2 threshold in current guidelines. However, the optimal SpO2 threshold that balances safety and effectiveness is not well established in children. Due to the lack of high-quality evidence, SpO2 thresholds for supplemental oxygen vary between (inter)national guidelines and hospitals and typically range from 90% to 94%.^4^,^6^

A 2014 Cochrane systematic review on supplemental oxygen therapy in children with lower respiratory tract infections revealed no studies on safe and effective SpO2 thresholds.^7^ In children up to 12 months with bronchiolitis, a randomised controlled trial has shown the safety of a 90% threshold compared with a 94% threshold.^8^ Children in the 90% saturation threshold group had an equivalent duration of cough and a faster return to normal health according to parents and were likely to be discharged 10 hours earlier than those in the 94% threshold group. Other studies on children with respiratory distress have shown similar effectiveness while maintaining safety with lower SpO2 thresholds ranging from 80% to 88%.^9^,^11^ These studies varied in quality and settings. A systematic review concluded that an 88% threshold deserves further study as a potentially effective and safe threshold.^12^ It is likely that the commonly applied SpO2 threshold of 92% is too high, resulting in children receiving too much oxygen, too often and for too long causing unnecessary and prolonged hospitalisations and increasing the burden of disease for these children and their parents.

Given the variation in guidelines and clinical practice, the lack of high-quality evidence and the significant potential benefits for patients and their parents, studies on effective and safe SpO2 thresholds for children with respiratory distress are urgently needed. This study aims to investigate whether an SpO2 threshold of 88%, compared with 92%, in children aged 6 weeks to 12 years admitted with respiratory distress, results in a safe reduction in length of hospital stay and is cost-effective from a societal perspective.

## Methods and analysis

### Trial design

This multicentre, open-label, randomised controlled trial with two parallel arms compares an 88% SpO2 threshold for starting and stopping supplemental oxygen (intervention group) with a 92% SpO2 threshold (control group). Data will be collected during admission and via digital questionnaires 7, 28 and 90 days after discharge (see table 1, participant timeline).

**Table 1:** SPIRIT patient timeline

Time point	Enrolment	Allocation	Postallocation				
	−t_1_	0	Admission	Discharge	+7 days	+28 days	+90 days
Enrolment:							
Eligibility screen	X						
Informed consent	X						
Allocation		X					
Interventions:							
Intervention (88%)			X				
Control (92%)			X				
Assessments:							
Baseline			X	X			
Primary outcome: time to meeting all discharge criteria			X	X			
Length of stay, oxygen therapy, clinical characteristics			X	X			
PICU admission			X	X		X	
Readmissions or reassessments						X	
Recovery, duration of cough, duration of dyspnoea, time to return to school/daycare					X	X*	X*
Quality of life				X	X	X	X
Paediatric global health				X	X	X	X
Parental anxiety				X	X	X	
Cost-effectiveness			X		X		X

* Only if a patient has not recovered at +7 days time point.

PICUpaediatric intensive care unitSPIRITStandard Protocol Items: Recommendations for Interventional Trials

### Study setting

The trial will be conducted in the general paediatric wards of 10 general hospitals throughout the Netherlands. All sites are listed on the study website.^13^

The study commenced on 1 December 2022. The recruitment start date was 7 September 2023. The planned recruitment end date is 30 November 2025, and the trial end-date is planned for 1 December 2026.

### Participants

#### Inclusion criteria

To be eligible to participate in this study, a subject must meet all of the following criteria:

- 6 weeks to 12 years of age.
- Being hospitalised with respiratory distress due to bronchiolitis, viral wheeze or lower respiratory tract infection.
- Requiring supplemental oxygen as per usual care (SpO2<92% or on clinical indication as determined by the treating physician).

As a pragmatic approach, diagnoses are determined by the treating physician. They are free to use diagnostic tools and/or criteria as per usual care. The diagnosis of lower respiratory tract infection is any lower respiratory tract infection other than bronchiolitis. This can be a clinical diagnosis, not necessarily confirmed by chest radiograph or point-of-care ultrasound. In the case of overlapping diagnoses, the most prominent diagnosis is registered. If the diagnosis is changed during admittance, the diagnosis at enrolment will be used.

#### Exclusion criteria

A potential subject will be excluded from participation in this study if they meet any of the following criteria:

- Children with pre-existing cardiopulmonary, neurological or haematological conditions (eg, congenital thoracic malformation, airway malacia, postinfectious bronchiolitis obliterans, childhood interstitial lung disease, primary immune deficiency or neuromuscular comorbidity).
- Children born <32 weeks gestational age.
- Children already included in other studies, which potentially interfere with this study.
- No stable internet access (needed for answering online questionnaires).
- No command of Dutch or English language.
- Children previously included in the current study.

As respiratory distress in children with an asthma attack is mainly driven by hypoxia, they are at risk of undertreatment in the acute phase of the attack.^14^ Therefore, children aged 6–12 years of age experiencing an asthma attack are excluded from this study.

### Interventions

#### Intervention group

Patients in the intervention group will receive supplemental oxygen if:

- Their SpO2 levels drop below 88%, by:
  - 1%–2% for at least 15 min continuously.
  - >2% for any amount of time.
- When indicated by the treating physician for clinical symptoms.

#### Control group

Patients in the control group will receive supplemental oxygen if:

- Their SpO2 levels drop below 92%, by:
  - 1%–2% for at least 15 min continuously.
  - >2% for any amount of time.
- When indicated by the treating physician for clinical symptoms.

Supplemental oxygen can be administered by either a face mask or nasal cannula, following local protocols. Patients are weaned of supplemental oxygen according to local protocols and based on the randomised SpO2 threshold.

Patients transferred to a paediatric intensive care unit (PICU) will receive standard care according to local PICU protocols, including SpO2 thresholds, and will be analysed according to protocol and included in the safety analyses.

To ensure adherence to the study protocol, both nurses and physicians at each centre will be trained to follow the study protocol. Any deviations from this protocol must be documented. Study protocol adherence is monitored by the registration of start and stop times of supplemental oxygen administration, including the reasons for starting and stopping, and clinical symptoms of the patient such as respiratory rate, heart rate and work of breathing. All other care is as usual. There are no prohibited concomitant care activities, drug prescriptions or interventions.

### Randomisation and allocation procedure

Patients are randomly assigned to either an 88% SpO2 threshold or a 92% SpO2 threshold. Allocation is done by the treating physician or nurse by drawing an opaque sealed envelope after obtaining written informed consent. The randomisation sequence is generated using a computer random number generator by a research team member not involved in recruitment or patient allocation. The sequence is based on a 1:1 block randomisation sequence, using varying block sizes, and is stratified for study site and age group (6 weeks to 1 year; 1 to 4 years and 4 to 12 years). Due to the nature of the intervention, participants, treating physicians and research team members cannot be blinded to the allocation. Although altering devices as done in the study of Cunningham *et al*^8^ would be ideal, device manufacturers are no longer willing to allow for such modifications due to tightened EU medical Devices Regulation requirements and liability concerns.

### Outcomes

#### Primary outcome

The primary outcome of this trial is the time from admission to when all prespecified discharge criteria are met. Prespecified discharge criteria are:

- No need for supplemental oxygen for 4 hours, including a period of sleep for children aged <2 years.
- Clinically fit for discharge with normal or minimally increased respiratory rate AND no or mild respiratory distress, as judged by nurses and physicians using the Parshuram *et al*^15^ scoring system, commonly used in Dutch paediatric practice as part of the Paediatric Early Warning Scale.^16^
- No need for in-hospital feeding or medication by nasogastric tube.
- No need for in-hospital intravenous treatment.
- No need for in-hospital nebulised bronchodilator treatment.
- No need for in-hospital treatment with metered dose inhalator inhalations more often than every 3 hours.
- No need for high flow delivered by high flow nasal cannula or nasal prongs.

Nursing staff continuously monitor discharge criteria and register them when met. Clinical fitness for discharge is checked every 4 hours, starting 4 hours after oxygen therapy is first stopped. If oxygen therapy is not initiated, this criterion is checked every 4 hours starting 4 hours after randomisation. In clinical practice, the actual discharge time may be later than when all discharge criteria are met. The reasons for any delay in discharge will be documented.

#### Secondary outcomes

Effectiveness:

- Length of hospital stay, measured in hours from admission to discharge.
- Time spent on oxygen therapy, measured in hours.

#### Safety outcomes

- Number of PICU admissions.
- Number of unscheduled readmissions and revisits to healthcare providers, as reported by parents in follow-up questionnaires and registered from patient electronic health records.
- Duration of symptoms, defined as time in days from admission to meeting the following criteria:
  - Resolution of cough (less than one cough every hour), as reported by parents.
  - Resolution of dyspnoea, as reported by parents.
  - Cessation of scheduled salbutamol use, as reported by parents.
- Time in days from admission to return to normal health, as reported by parents.
- Time in days from admission to return to school/day care, as reported by parents.
- Patient quality of life during follow-up, measured by digital questionnaires at discharge, 7 days, 28 days and 90 days after discharge by EuroQol Five Dimensions Health Questionnaire Youth (EQ-5D-Y) (by patient or by parent proxy form). For ages 6 weeks to 2 years, only the EuroQol Visual Analogue Scale (EQ-VAS) is used, as this is the only tool with good clinimetric properties in this age group which can be compared with the full 6 weeks to 12 years age range.^17^ For ages 2 and 3 years, a version of the EQ-5D-Y with modified wording is used, developed in an Australian study (currently in submission) and has been translated with the help of the EuroQol offices. For ages 4–12 years, the EQ-5D-Y is used (proxy version for up to 8 years, self-complete version for 8–12 years), validated in this age group.^18 19^
- Parental anxiety up to 1 month after discharge, measured by digital questionnaires at discharge, 7 days and 28 days after discharge by the anxiety items on Hospital Anxiety and Depression Scale.^20^
- Overall paediatric health was measured by digital questionnaires of International Consortium for Health Outcome Measurement (ICHOM) Patient-Reported Outcomes Measurment Information System (PROMIS) set at discharge, 7, 28 and 90 days follow-ups.

#### Economic evaluation

The economic evaluation aims to relate the incremental costs of an SpO2 threshold of 88% (intervention) with an SpO2 threshold of 92% (control) to the incremental health effects. Both cost-effectiveness analysis and a cost–utility analysis will be performed from a societal and healthcare perspective according to Dutch guidelines with a time horizon of 90 days postdischarge.^21^ Discounting is not necessary as the time horizon is less than 12 months. Costs will be measured from a societal perspective using web-based questionnaires based on the iMTA Medical Consumption Questionnaire and iMTA Productivity Cost Questionnaire at discharge and 90 days after discharge.

Cost categories include:

1. Healthcare costs (primary/secondary/tertiary care, complementary care and home care).
2. Lost productivity costs of the parents (absenteeism from paid and unpaid work).
3. Patient costs (informal care and other care services paid for by the patients themselves).

Valuation follows Dutch cost guidelines,^22^ with absenteeism from paid work using the friction cost approach. Effect measures in the economic evaluation will include:

1. Length of hospital stay.
2. Overall paediatric health by ICHOM PROMIS set.
3. Parent anxiety.
4. Quality-adjusted life-years (EQ-5D-Y with Dutch reference values).^23^

#### Sample size and feasibility

The sample size calculation is based on the mean time to meet all discharge criteria for the two groups. We aim to demonstrate a difference of 12 hours, determined as a relevant reduction through patient/parent interviews and supported by previous research.^8^ With a mean length of stay of 72 hours and an SD of 48 hours (based on 5 years of admissions data for respiratory distress at our hospital, Statistics Netherlands (Centraal Bureau voor Statistiek (CBS)) data on admission duration and previous research^8 9^) an alpha of 0.05 and 80% power, 251 children are to be included in both intervention and control groups. With an estimated dropout rate of 10%, the goal is to include 560 patients over the 30-month inclusion period. This is feasible, as two participating centres admit an average of 200 children aged 0–12 years with respiratory disease per year. Estimating 80% eligibility, 15% not approached and 60% willingness to participate (based on parent interviews, in-hospital data and expert estimation), and with 10 participating, centres over 30 months up to 2000 children could be included.

#### Patient screening and recruitment

Potential subjects and their parents/caregivers will first be informed using posters at the emergency department. Eligible subjects and their families will be screened by the attending physician and, if eligible, will be asked for informed consent by either their attending physician or an independent study nurse or doctor (see informed consent form, online supplemental file 2).

**Figure 1:**
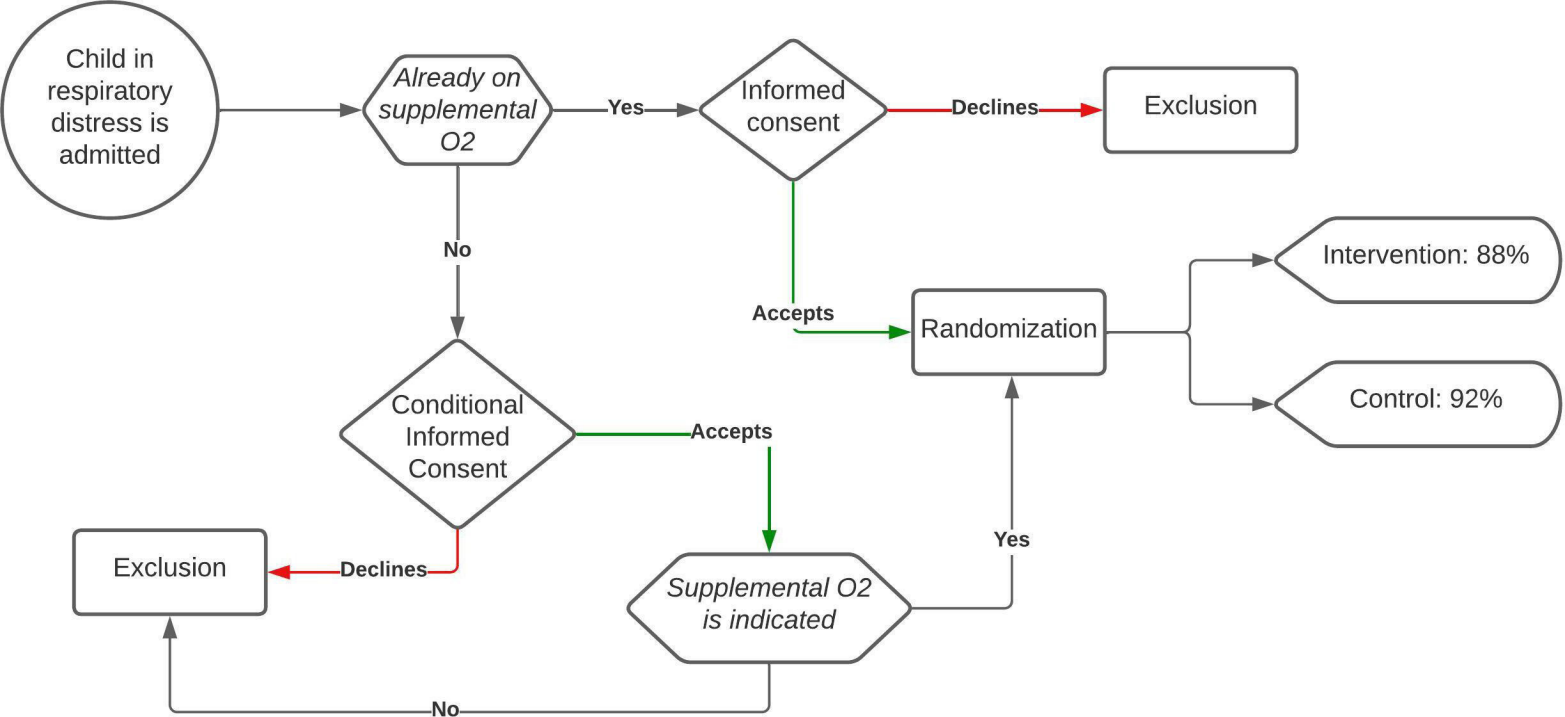
Informed consent flow chart.

Conditional informed consent: For patients meeting all inclusion criteria at admission but not (yet) in need of supplemental oxygen, parents/guardians will be asked to sign informed consent conditionally. If the need for supplemental oxygen arises, consent is verbally confirmed and documented in the medical chart. If no supplemental oxygen is indicated during admission, the patient will not be included in follow-up and no additional data will be collected (see figure 1).

Study initiation: Study procedures begin when at least one parent/guardian provides written informed consent and the second has given verbal consent. Written consent from the second parent/guardian will be obtained within 72 hours after inclusion. The procedure is designed to accommodate emergency situations, where one parent is often present, ensuring the intervention can start as early as possible.

Informed consent process: Adequate time is provided for the informed consent process, allowing parents/guardians to receive information, ask questions, consult with family and consider their decision. For patients already on supplemental oxygen, study enrolment must be completed within 6 hours of the start of oxygen therapy to minimise time spent on supplemental oxygen outside the study protocol. For patients not yet on supplemental oxygen, informed consent is ideally obtained before the need arises but must be completed within 6 hours after the start of oxygen therapy. Beyond this timeframe, children are no longer eligible for participation.

#### Data collection

Attending nurses will fill out data collection forms for each subject during admission. These forms will be used to enter data in a digital data capture programme (Research Manager). Once a subject is registered in Research Manager, digital questionnaires are automatically sent at the follow-up time points, with reminders after 1 week. If unanswered, patients will be contacted by telephone or text message to be reminded or to complete essential safety data questions by phone. Pseudonymisation is used to code the collected data, with only the local research team having access to the pseudonymisation key.

#### Data management and archiving

Data will be managed based on a data management plan that has been established for this project. Data will be collected and processed following the General Data Protection Regulation (EU) 2016/679. The clinical trial master file’s content, including data and source documents, will be archived for at least 25 years after the clinical trial’s conclusion.

#### Statistical methods

##### Primary study parameter(s)

The time to meet all discharge criteria will be presented in hours. The primary analysis will be conducted on an intention-to-treat basis. Point estimates and uncertainty will be chosen based on the distribution of the data. The difference in hours between the groups will be analysed and tested for statistical significance, adjusting for centre and age and using a multilevel linear regression model for hospitals, with diagnosis and patient clinical parameters at presentation as covariates. A 12-hour difference is deemed clinically significant. Sensitivity analysis will include a per-protocol analysis of the primary outcome. For other time-to-event parameters, a Cox proportional hazards model will be used to estimate the intervention effect.

##### Missing data

- If the time to meet all discharge criteria is missing because the time of meeting any/all discharge criteria was not noted, the point-estimate difference between the time of meeting all criteria and the time of discharge in the recorded population will be subtracted from the time of discharge and used instead.
- If time to meeting all discharge criteria are missing because the child was transferred to the PICU, these patients are excluded from the primary analysis but included in the safety analyses.
- Missing data in the covariates will be investigated to identify the mechanism (missing not at random, at random or completely at random) and handled appropriately. The default approach will be to use multiple imputation with all data available on the subjects. A complete-case analysis will be performed as a sensitivity analysis.

##### Secondary study parameter(s)

All analyses of secondary study parameters will use an intention-to-treat approach, with a per-protocol approach performed as a sensitivity analysis. All point estimates will be presented according to the distribution of the data (mean/median with standard deviation (SD) / Interquartile Range (IQR)).

- Count outcomes: For count outcomes like PICU transfers, readmission and unscheduled visits to healthcare providers, multilevel Poisson regression will be used.
- Oxygen therapy duration: To investigate differences in time on oxygen therapy between groups, multivariable linear regression will be applied.
- Time-to-event outcomes: For outcomes such as time to last day of cough and/or salbutamol use, time to normal activities and time to discharge, Cox proportional hazards models will estimate the treatment effect. Time-to-event data will be either interval-censored or right-censored. All models will be adjusted for hospital, age, gender, respiratory distress severity at baseline, diagnosis and smoking by parents/patients if possible.
- Quality of life, anxiety measures and ICHOM PROMIS scores are continuous repeated measures and will be analysed using multilevel models, with random effects accounting for multiple measurements within each individual.
- Exploratory subgroup analyses: These will be based on diagnosis, age and skin type, as skin type might influence the accuracy SpO2 measurement.^24^,^26^ Skin type will be recorded in the discharge questionnaire by the parent as one of the six skin types defined by Fitzpatrick.^27^

##### Other study parameters

Patient demographics and baseline characteristics will be presented using n (%) for count and nominal data, and mean/median and SD/IQR for continuous data.

- Reasons for oxygen therapy: The reasons for starting and stopping oxygen therapy are nominal data. Differences between groups will be tested using regression techniques if applicable.
- Patient condition: The patient’s condition at the start and stop of oxygen therapy, and at discharge, will be presented as individual parameters, standardised for age where necessary.
  - Heart rate, respiratory rate and SpO2 are continuous data.
  - Chest retractions are ordinal data, reporting the extent and severity (jugular, subclavicular, intercostal and subcostal).
- Statistical tests: Parameters will be tested for differences between the groups by appropriate statistical tests (t-test/Mann-Whitney U test/analysis of variance).

##### Statistical analysis for the economic evaluation

All statistical analyses will be conducted based on the intention-to-treat principle. Missing cost-and-effect data will be imputed using multiple imputations according to the Multivariate Imputation by Chained-Equations (MICE) algorithm. Rubin’s rules will pool the results from the different multiply imputed datasets. Linear regression analyses will estimate cost-and-effect differences between intervention and control groups, adjusting for confounders if necessary.

Incremental cost-effectiveness ratios (ICERs) will be calculated by dividing the difference in the mean total costs between treatment groups by the difference in mean effects between treatment groups. Bias-corrected and accelerated bootstrapping with 5000 replications will be used to estimate 95% CIs around the cost differences and statistical uncertainty surrounding the ICERs.

Uncertainty surrounding the ICERs will be graphically presented on cost-effectiveness planes. Additionally, cost-effectiveness acceptability curves will be estimated to show the probability that the intervention is cost-effective compared with the control, across a range of different ceiling ratios, thereby illustrating decision uncertainty.^28^

##### Study procedures

###### Monitoring

An independent data safety and monitoring board (DSMB) will monitor this trial, composed of experts in paediatrics, epidemiology and statistics. Despite the trial being classified as having negligible risk, the involvement of minors incapable of giving informed consent necessitates the DSMB’s existence. The DSMB Charter outlines the board’s composition, statement on interim analysis, stopping guidelines, conflict of interests declarations and a summary of tasks and responsibilities.

An independent monitor will conduct an on-site visit at each participating centre during the trial to review the informed consent procedure, inclusion and exclusion adherence and source data verification.

### Procedures for recording and reporting adverse events

In this low intervention trial, procedures for recording and reporting of (severe) adverse events ((S)AEs) are based on a risk-proportionate approach. They follow recommendations from the Dutch Central Committee on Research Involving Human Subjects (CCMO).

Recording AEs: All AEs reported spontaneously by the subject, his/her parent(s)/guardian(s) or observed by the investigator or his staff will be recorded up until 28 days of follow-up or until the end of the disease, whichever comes last, but no longer than full study follow-up.

Exceptions include:

- Elective hospital admissions or procedures.
- Common symptoms of bronchiolitis, viral wheeze or lower respiratory tract infections, such as coughing, dyspnoea, rhinitis, fatigue, sleep disturbance, food intolerance and bronchodilator requirement. A selection of these events (duration of cough, duration of dyspnoea and bronchodilator use) are recorded and reported within the patient questionnaires as safety parameters.
- Deterioration in respiratory symptoms during admission, identified by the need for high-flow nasal cannula or CPAP, is recorded and reported within the electronic Case Report Form (e-CRF).
- Hospital readmissions (SAE) or reassessments (AE) are recorded and reported within the e-CRF.

Immediate reporting (within 24 hours): The following SAEs are critical to safety evaluations and require immediate reporting from the investigator to the sponsor:

- PICU admissions.
- SAEs that result in persistent disability/incapacity or death.
- Other SAEs likely related to the study intervention, such as readmissions within 28 days.

Non-reporting SAEs: No reporting is required for the following (S)AEs as no causal relationship with the study procedure is expected:

- AEs and SAEs occur after signing informed consent but before patient randomisation or initiation of the assigned SpO2 threshold.
- SAEs not part of the listed SAEs requiring reporting and not considered Suspected Unexpected Serious Adverse Reaction (SUSAR).

Follow-up on AEs: All AEs will be followed until they have been resolved or until a stable situation has been reached. Follow-up may require additional tests or medical procedures as needed and/or referral to a general physician or a medical specialist.

## Ethics and dissemination

The study will be conducted according to the principles of the Declaration of Helsinki (version 2013) and in accordance with the Medical Research Involving Human Subjects Act (WMO). It has been approved by the research ethics committee (REC) of Leiden, Den Haag, Delft (EU CT number 2023-504817-56, REC number P23.045). Any important protocol modifications will be communicated to the REC and regulators.

### Patient and public involvement

Patients and/or their representatives were involved in the design of this trial and will remain involved during all phases. They were recruited with the aid of the foundation ‘Child and Hospital’ and the Lung Foundation of the Netherlands, along with a focus group of parents and patients who were admitted to the general ward with respiratory distress of the Spaarne Gasthuis. Their input will be sought when interpreting the results and when drafting lay publications.

#### Access to data

Data will be published in accordance with ZonMw’s FAIR data standards for Open Access publication, as described in the Data Management Plan.

#### Dissemination

The results of this trial will be shared through lay publications, scientific journals and academic conferences. Authorship will follow the guidelines defined by the International Committee of Medical Journal Editors (http://www.icmje.org).

## Discussion

Safe and effective oxygen therapy in children is crucial, as both undertreatment and overtreatment can have serious consequences. However, the optimal SpO2 threshold that balances safety and effectiveness is not well established in children. The main goal of the OxyKids trial is to investigate whether an SpO2 threshold of 88%, compared with 92%, in children aged 6 weeks to 12 years admitted with respiratory distress, results in a safe reduction in length of hospital stay. It uses a pragmatic design with procedures closely resembling daily clinical practice. Together with the broad range of ages and varying diagnoses, generalisability of the findings is high and implementation is facilitated.

The open-label design of the trial introduces a risk of bias that is difficult to overcome without manipulating pulse oximeter equipment, a procedure that manufacturers did not want to facilitate. To mitigate this risk all study personnel, clinicians and nurses undergo thorough training to follow the study procedures. Additionally, to quantify any potential bias, clinical parameters such as respiratory rate, heart rate and work of breathing at start and stop of supplemental oxygen will be recorded.

Due to the pragmatic design, children in the OxyKids trial will have continuous SpO2 monitoring. As was shown previously, hypoxaemia can occur in sleep without clinical deterioration in young children with bronchiolitis.^29^ Moreover, continuous SpO2 monitoring may unnecessarily escalate treatment and prolong hospitalisation in infants with bronchiolitis.^30^ However, as the OxyKids trial compares different saturation thresholds within the current standard of care in both children with bronchiolitis and older children with other diagnoses, limiting SpO2 measurements only to awake state is not in line with the aim of the trial, even though doing so could be the better clinical practice.

A common concern with lowering SpO2 thresholds is the potential for long-term neurocognitive sequelae.^31^ Children with congenital heart disease or sleep-disordered breathing and chronic or intermittent hypoxaemia in the low 90% range also had adverse cognitive outcomes.^32^ The question remains if these results can be extrapolated to a population of otherwise healthy children with acute short-term hypoxaemia. Similar to other trials investigating the safety of lower thresholds, the OxyKids trial describes short-term follow-up. The follow-up of long-term neurocognitive outcomes poses significant challenges, including standardised assessments of neurocognitive outcome measures in varying age groups, and assessment of long-term confounders. Nonetheless, follow-up studies are warranted to address these concerns.

The OxyKids trial results are expected to significantly influence clinical practice. This trial is expected to contribute to more standardised and evidence-based guidelines and provide greater insight into optimal SpO2 thresholds in paediatric medicine.

## supplementary material

10.1136/bmjopen-2024-087891 online supplemental file 1

10.1136/bmjopen-2024-087891 online supplemental file 2

10.1136/bmjopen-2024-087891 online supplemental file 3
