# Supplementary material for: Safe, effective and cost-effective oxygen saturation targets for children and adolescents with respiratory distress: protocol for a randomised controlled trial (OxyKids study)
Source: BMJ Open. 2024 Dec 22;14(12):e087891. doi: 10.1136/bmjopen-2024-087891 (PMC11667372; doi:10.1136/bmjopen-2024-087891)
Supplement: online supplemental file 1 [file bmjopen-14-12-s001.pdf]

Erasmus MC
Universitair Medisch Centrum Rotterdam

Data Management Plan

Please read the manual for guidance and examples!

#0 DMP details

0.1	Version of DMP	1.1
0.2	Name of person writing this DMP	Sam Louman
0.3	ORCID iD of person writing this DMP	0000-0002-3157-1223
0.4	Function of person writing this DMP	Arts-onderzoeker / PhD
0.5	Role in project of person writing this DMP	Project coordinator/ PhD
0.6	Data management support staff consulted?	☒ Yes:<br>☐ No
	If Yes: Describe the support team, name, email address and date of conversation	Sabrina Meertens-Gunput, RDM support team ErasmusMC. [redacted]<br>Date of conversation: 08-09-2022
0.7	Name of Principal Investigator	Dr. Annemie L. M. Boehmer
0.8	ORCID iD of Principal Investigator	0000-0002-3157-1223
0.9	Function of Principal Investigator	Project Leader

0.10	Date of authorisation by Principal Investigator	08-06-2023

#1 Project details

This section covers the general information about the project. You can copy this information from the grant application or other existing information about the project.

1.1	Full project title	Safe, effective and cost-effective saturation targets in children and adolescents with respiratory distress: a randomized controlled trial.
1.2	Acronym	OxyKids
1.3	Department of the PI	Kindergeneeskunde, Spaarne Gasthuis
1.4	PaNaMa project ID *(mandatory for all research involving human subjects)*	Will be applied for
1.5	Funder	☒ Funder: ZonMw<br>Grant #: 10390012110075<br>☐ None
1.6	(Planned) data collection start date	01-09-2024
1.7	(Planned) data collection end date	31-12-2025
1.8	Intention to describe the study results in a manuscript for publication in a journal	☒ Yes<br>☐ No
1.9	Research type(s)	Clinical, randomized controlled trial
1.10	Research objectives	Main objective: to investigate if a 88% SpO2 threshold for dyspnoeic children results in a safe reduction of length of stay, compared with 92%.<br>Secondary objectives: to investigate if the lower thresholds leads to differences in length and severity of symptoms, readmissions, time to normal activity, patient and parent quality of life and cost-effectiveness.
1.11	Endpoints	Main: time from admission to meeting all discharge criteria in hours.<br>Secondary:<br>- Length of stay, measured as time from admission to discharge in hours<br>- Number of PICU admissions per group<br>- Time spent on oxygen therapy in hours<br>- Duration of symptoms as defined by the study team. Measured as days from admission to having met all of the following criteria:

	o Resolution of cough (less than 1 cough every hour)<br>o Cessation of scheduled salbutamol inhalations<br>Upon discharge parents are asked to note the last day of cough and/or salbutamol use and report this during follow-up contacts.<br>- Return to normal health as reported by parents. Measured as days from admission to parent reported normal health. Upon discharge parents are asked to record the last day of illness and report this during follow-up contacts.<br>- Unscheduled health care reattendence or readmissions. Measured as the amount of visits or admissions to a health care provider for respiratory complaints during follow-up within 28 days after admission, as reported by parents.<br>- Patient quality of life on discharge, 7 days follow-up and 90 days follow-up, measured by EQ-5D-5L (by patient or by parent proxy form)<br>- Parent anxiety on discharge and 7 days follow-up measured by anxiety items on Hospital Anxiety and Depression Scale (HADS).<br>- Overall pediatric health measured by ICHOM PROMIS set at discharge, 7 days follow-up and 90 days follow-up.

#2 Agreements and intellectual property

This section covers the involved parties and the agreements that were made with these parties, and intellectual property.

2.1	Single centre / multicentre study	☐ Single centre → 2.3<br>☒ Multicentre
2.2	Role of Erasmus MC<br>*(if multicentre)*	☐ Initiating centre<br>☐ Initiating centre as part of consortium<br>☒ Participating centre
2.3	Involvement of external parties and their roles<br>*Please explain details below.*	☐ Consortium partner(s):<br>*(if Erasmus MC is part of consortium)*<br>☒ Participating centre(s): see below<br>*(if Erasmus MC is initiator / part of consortium)*<br>☒ Initiating centre(s): Spaarne Gasthuis<br>*(if Erasmus MC is participating centre)*<br>☒ Sponsor in a Clinical Trial: ZonMw<br>☐ Parties consulted for reuse of pre-existing data:<br>☐ Parties consulted for data processing:<br>☐ Other parties for other purposes:<br>☐ None
	If external parties are involved:<br>Describe which agreements were made, with which party, which party initiated the agreement and whether these have been written or checked by the PCP, TTO and/or PKO (e.g., CTA, DTA, MTA, DPA).	Other participating centres are currently:<br>- Martini<br>- CWZ<br>- Isala<br>- Amphia<br>- Tergooi<br>- St. Antonius<br>- Haaglanden MC<br>- Franciscus<br>- Rijnstate<br><br>The Spaarne Gasthuis initiated a Consortium Agreement and Sponsor Agreements. Other agreements are yet to be made. In the Consortium Agreement clear agreements have been made regarding intellectual property. Data sharing will be described in separate DTAs.
2.4	Describe how ownership and intellectual property rights are managed.	Ownership and intellectual property agreements have been made in the Consortium Agreement.

#3 Ethical and legal issues

This section addresses ethics review and requires you to confirm your awareness of and compliancy with the applicable laws and regulations.

3.1	Does this project involve data and/or biological material acquired from humans?	☒ Yes<br>☐ No → 3.5
3.2	Is this study subject to the WMO or Wbo?	☐ Non-WMO → 3.4<br>☒ WMO<br>☐ Wbo
3.3	Was this proposal approved by a MREC/METC, the CCMO or the RIVM/CvB?	☒ Yes, by the METC<br>☐ Yes, by the CCMO<br>☐ Yes, by the RIVM/CvB<br>☐ Not yet:<br>→ 3.8
3.4	Did the MREC/METC provide a non-WMO-declaration?	☐ Yes<br>☐ Not yet:
3.5	Does this project involve data and/or biological material acquired from animals?	☐ Yes<br>☐ No → 3.8
3.6	Was this proposal approved by the DEC and was a permit received from the CCD?	☐ Yes<br>☐ Not yet:
3.7	PreclinicalTrials registration number *(if animal research)*	

3.8 Please confirm your awareness of and compliancy with the applicable laws and regulations for this project:

Applies to:

Personal data	☒	🇬🇧 General Data Protection Regulation (GDPR)<br>🇳🇱 Algemene Verordening Gegevensbescherming (AVG)
Patient care data	☒	🇬🇧 Medical Treatment Contracts Act<br>🇳🇱 Wet op de Geneeskundige Behandelingsovereenkomst (WGBO)
WMO	☒	🇬🇧 Medical Research Involving Human Subjects Act<br>🇳🇱 Wet Medisch-Wetenschappelijk Onderzoek met mensen (WMO)
	☒	🇬🇧 Guideline Quality Assurance of Research Involving Human Subjects<br>🇳🇱 Richtlijn Kwaliteitsborging Mensgebonden Onderzoek
Wbo	☐	🇬🇧 Population Screening Act<br>🇳🇱 Wet op het Bevolkingsonderzoek (Wbo)
Non-WMO	☐	🇬🇧 Code of Conduct for Health Research<br>🇳🇱 Gedragscode Gezondheidsonderzoek (Code Goed Gedrag)
	☐	🇬🇧 Code of Conduct for Responsible Use of Human Tissue<br>🇳🇱 Gedragscode Goed Gebruik van Lichaamsmateriaal (Code Goed Gebruik)
Human embryos	☐	🇬🇧 Embryo Act<br>🇳🇱 Embryowet
Animals	☐	🇬🇧 Act on Animals used for Scientific Purposes<br>🇳🇱 Wet op de Dierproeven
All	☒	🇬🇧 Erasmus MC Research Code<br>🇳🇱 Erasmus MC Research Code
	☒	🇬🇧 Netherlands Code of Conduct for Research Integrity<br>🇳🇱 Nederlandse Gedragscode Wetenschappelijke Integriteit
	☒	**I confirm** that I am aware of and compliant with the laws and regulations as indicated above, and any relevant supplementary laws and/or regulations, e.g., for research involving medicinal products, medical devices and/or in vitro diagnostics

#4 Informed consent

This section covers information about informed consent.

4.1	Is there a form of informed consent?	☒ Yes, CCMO format<br>☐ Other (e.g., partly, indirectly):<br>☐ No:<br>☐ N/A (no research involving human subjects) → 5.1
	If Other: Describe the situation.	
	If No: Explain why this project involves the collection, processing or analysing of personal data without informed consent and describe whether this was discussed with PKO.	
4.2	Describe the recruitment and informed consent procedure.	Potential subjects are identified by local clinicians at the emergency department upon presentation. The decision to admit the patient prompts the informed consent procedure. A local clinician or investigator, all trained for the informed consent procedure, will inform the subject of the study, and/or their parents/caretakers, and ask for their consent upon admission. Subjects and/or their parents/caretakers will have up to 4 hours to consider their decision.
4.3	The following GDPR/AVG participant rights apply to scientific research:<br>• Right to be informed<br>• Right of access to copy of data and processing information<br>• Right to object<br>• Right to erasure<br><br>Describe what happens when participants make use of their GDPR/AVG rights and what procedures are put in place to ensure an appropriate response to their request.	Participants are informed about the processing of their data and their rights through the subject information sheet.<br>When a participant refuses to participate, their data will not be collected.<br>When a participant withdraws consent, the data collected up to the moment of withdrawal will be used (according to GDPR/AVG Article 89(1)), but no further data will be collected.<br>When a participant requests to receive their data, the identity of the person will be verified and the principal investigator will make a data extraction of the participant's data only and will send the data to the participant via an encrypted link.
4.4	Does this informed consent form provide for the data to be reused for future research?<br>*(if informed consent is asked)*	☒ Yes, via an optional question<br>☐ Yes, via a mandatory condition<br>☐ No → 5.1<br>☐ N/A → 5.1
4.5	By whom may data be reused according to the subject information sheet and/or informed consent form?<br>*(if question/condition about data reuse is present in informed consent form)*	☒ Own research group<br>☒ Other research institutes<br>☐ Commercial parties

#5 Data description

This section covers the description of the data that will be collected, used or generated during this project.

5.1	Which type(s) of data will be collected, (re)used and/or generated during this project?	☒ Quantitative data (e.g., databases, spreadsheets)<br>☐ Qualitative data (e.g., images, audio, video, text)<br>☐ Biological material (e.g., blood, tissue)<br>☐ Other:
5.2	Will pre-existing healthcare data from the Electronic Health Record (EHR or in Dutch: EPD) be used?	☐ Yes:<br>☒ No
	If Yes: Describe which (kind of) data will be extracted and from which source(s).	
5.3	Will any other pre-existing data be used?	☐ Yes:<br>☒ No → 5.5
	If Yes: Describe which (kind of) data will be reused and from which source(s).	
5.4	Describe how and by whom permission was granted to reuse the data and describe the terms of use (if applicable).	
5.5	Will new data be collected or generated during this project?	☒ Yes:<br>☐ No
	If Yes: Describe which (kind of) data will be collected or generated.	We will collect new data on patient demographics, health states, quality of life, anxiety and cost-effectiveness.
5.6	Describe how the data will be collected, (re)used or generated for this project, including for each data source: the origin, the sample size, the data collection tool or method and the file format (if applicable).	Patient details of 560 patients divided over all participating centres will be collected by eCRF in Research Manager and extracted in csv format.<br><br>We will use Research Manager to send surveys to study subjects with health state questions, quality of life and anxiety questionnaires and additional questions on costs made. We have permission to use the validated EQ-5D-Y, Hospital Anxiety and Depression Scale and PROMIS Core Outcome Set Pediatric Global Health. Data will be extracted from Research Manager and provided in csv-format.
5.7	Describe how the data described above will be processed, combined and/or (re)used, including the tools and file formats that will be used.	Data will be combined and processed using R, R Studio. This will be done in R Markdown files.

5.8	Describe how the data quality control will be ensured.	We will use a certified data capture tool: Research Manager, which will use skips and validation checks. A transparent audit trail will be available. A data monitor will perform source data verification on the entered data.<br><br>We will check the dataset for completeness, correctness, duplicates, uniformity and consistency using R. If as result of the dataset checking the data need to be cleaned, we will apply corrections to the data in Research Manager or R in a traceable way.<br><br>Raw data will be locked when all data validation checks are completed to avoid (accidental) modifications of the raw data.<br><br>Generated syntaxes/codes for processing and/or analysis in R are reviewed by a statician.
5.9	Does this project require personal data (either directly or indirectly identifiable data) for patient selection, data collection, data processing and/or analysis?	☒ Yes:<br>☐ No → 5.13
	If Yes: Describe which personal or identifiable data will be collected and explain why.	Patient ID will be collected to be able to trace back to the patient.<br>Contact details (name, email address and phone number) are required to send surveys and follow-up.<br>Age in years and gender are required to describe the study population.<br>Clinical data is required to answer the research question.
5.10	Describe whether, when, how, by whom and which data will be pseudonymised and/or anonymised during the project.	The research data will be pseudonymised by the research coordinator as soon as the first record of the participant is created in the research database. The data that is entered in the database does not contain any directly identifying data. This way, the identity of the subject is only to be re-identified by using the pseudonymisation key. This key is stored separately from the data and is managed by the Project Coördinator and Project Leader. After the project is completed, only the Project Leader will have access to the pseudonymisation key file." When the Project Leader terminates their function, their responsibilities will be transferred to a new Project Leader within the Project Group.
5.11	Which other steps will be taken to safeguard the privacy and confidentiality of the participants and	We have completed a full DPIA to assess the privacy risks of this project and took the required mitigating

	to ensure compliance with (institutional) legislation and regulation on personal data?	measures (please specify if not already listed in your answer).<br><br>All project team members have signed a non-disclosure agreement as part of the Consortium Agreement.<br><br>Participants will be asked to provide informed consent.<br><br>We will be using Research Manager to collect only these data variables that are necessary to answer our research question and no directly identifying personal data. As for the data values, we will limit the use of free text fields and use pre-defined answer options in order to limit unnecessary level of detail. Date variables that are only needed to perform calculations within Research Manager will not be exported, in order to limit potentially identifiable data in the output files. Email addresses will only be accessible to authorised users and will not be exported. The Research Manager study and data is only accessible for authorized study personnel.
5.12	Describe the internal procedure in case of a possible data breach.	In case of a data breach, we follow the rules for security incidents according to the center where the breach occurred. Usually this involves: inform the Privacy Contact Person; determine together if there is a data breach involving personal information; inform the department head or supervisor; and make an internal report.
5.13	Are facilities required for data collection and/or generation already present?	☒ Yes<br>☐ No:
	If No: Describe what still needs to be arranged.	eCRF and surveys need to be built in to Research Manager

#6 Standardisation and documentation

This section covers the standards and documentation methods that will be used to standardise the project information and data in order to improve the reusability.

6.1	Will a terminology standard or standardised form be used to standardise the data?	☒ Yes:<br>☐ No:
	Describe which terminology and/or form standard(s) will be used or explain why not.	We will be using SNOMED as a terminology standard for healthcare variables, and the ICD-10 code system to classify diagnoses. The EQ-5D-Y, Hospital Anxiety and Depression Scale and Pediatric Global Health Outcome Set by ICHOM PROMISE will be used as validated questionnaires in a standardised manner.
6.2	Describe how the data-level documentation will be described.	We will create a data dictionary spreadsheet to describe the dataset metadata.
6.3	Describe how the project-level documentation will be described.	We will describe our project-level documentation in our research protocol, data management plan, data validation plan and data analysis plan, so that another researcher would be able to reproduce our results.<br><br>We will document all our decisions regarding data processing and cleaning in comments in our R-scripts.
6.4	Describe how the folder structure, file naming conventions and version control will be arranged.	The folder structure will be organised logically for our research project and will contain ReadMe text files that include information on how the (meta)data and documentation are organised in the folder structure. We will mark the files with version numbers. Correct version control will be regulated by using a version control table. Every month, versions are reviewed and previous versions will be archived in an archive-folder.

#7 Data storage and backup

This section covers the data storage and backup during the project.

7.1	Give an estimation of the total amount of data that will be stored during this project.	☐ 0-10 GB<br>☒ 10-100 GB<br>☐ 100-1000 GB<br>☐ 1-100 TB<br>☐ >100 TB
7.2	Which storage location(s) will be used?	☐ Research storage & compute services<br>☐ Digital Research Environment (DRE)<br>☐ Isilon file services<br>☒ SharePoint<br>☐ V-drive<br>☐ Central Cold Storage via Central Biobank<br>☒ Other: Research manager and local inhospital drive.
7.3	Describe how access control of the storage location(s) is managed.	Access can be requested and granted by the PI, is logged in an access log. Access will only be granted to files necessary to the person's function within the study team. Only the local study team will have access to the local pseudonymisation key. Furthermore, relevant authorized parties (ie monitor, IGJ) will be granted access for monitoring or auditing purposes. Directly identifying data is stored in a different folder and will be password protected, only accessible by PI and PhD.
7.4	Describe the backup protocol (e.g., frequency, automatically or manually).	SharePoint, storage has automatic backups
7.5	Will data be transferred from or to the Erasmus MC?	☐ Yes:<br>☒ No
	If Yes: Describe how data will be transferred.	Data will be transferred from participating centres to Spaarne Gasthuis. Research Manager will be used as an electronic data capture tool, where each participating centre has access to their own data and Spaarne Gasthuis has access to all data.

#8 Data archiving

This section covers the data archiving after the project. Please note that archiving is mainly for compliancy and verification purposes. The archiving of data also enables sharing of the data for reuse, but not all the archived data is suitable for sharing. Data sharing will be covered in the next section.

8.1	Describe which criteria will be used to determine which data will be archived.	If the research data has great value, is unique, difficult or expensive to (re)produce or should be preserved for the long term due to obligations, the data will be archived. Data that can easily be reproduced (e.g., processed versions of data) will be destroyed, because this would cost too much archiving capacity and costs. Data or metadata with no use at all in the future will be destroyed (e.g., contact details). At least all raw and final data, used scripts and original paper documents with wet signatures will be archived.
8.2	Are there changes required to make the data and/or documentation readable and usable in the long term (e.g., conversion to preferred file formats)?	☒ Yes:<br>☐ No
	If Yes: Describe which changes will be made.	For the interoperability and reusability of the data, we will convert the used file formats to preferred file formats that are compatible with generally used software packages, in necessary.<br><br>Documentation files that were originally created in Word, will be saved as PDF files.
8.3	For which duration will the data be archived?	☐ At least 30 years (ATMPs)<br>☒ At least 25 years (medicinal products)<br>☐ At least 15 years (WMO)<br>☐ At least 10 years (non-WMO, non-implantable medical device)<br>☐ Other:
8.4	Where will the data be archived after the project?	☐ Research storage & compute services<br>☐ Isilon archive services<br>☐ Paper document storage via OASIS<br>☐ Central Cold Storage via Central Biobank<br>☒ Other: Iron Mountain for paper document storage. Digital documents will be stored on SharePoint.
8.5	Describe how access control of the archive will be managed.	Only the Principal Investigator (PI) and Science Bureau of Spaarne Gasthuis will have access to the archive. The PI is responsible for the access control of the archive and will handover this responsibility upon termination of employment.

#9 Data sharing

This section covers the data sharing and publishing after the project.

9.1 Do you consider the data relevant for reuse?

☒ Yes:
☐ No:

Describe for which researchers or research groups the accessibility of this data is important or explain why not.

For researchers within the field of (pediatric) pulmonology and/or cost-effectiveness.

9.2 Will the (meta)data (partly) be made available for reuse?

☒ Yes
☐ No: → 10.1

If No: Explain why not.

9.3 Are there additional changes required before the dataset can be shared (e.g., aggregation, filtering)?

☒ Yes:
☐ No

If Yes: Describe which changes will be made to the dataset before it will be shared.

Data from participants who did not grant permission to reuse their data will be removed. Identifiable data will not be shared.

9.4 Which data and other end products from this project will be made available for future research?

☒ Study protocol
☒ Data management plan
☒ Data validation plan
☒ Data analysis plan
☒ Raw data
☒ Processed data
☒ Final data
☒ Metadata
☒ Codebook
☒ Documentation
☒ Scripts to process data
☒ Scripts to analyse data
☒ Scripts to generate tables and figures in the publication
☐ Lab journals
☐ Biological material
☐ Audio-visual material
☒ ReadMe file with an overview of files and file content and use
☐ Other:

9.5 Does this project involve the use or generation of software that is required in order to access and interpret the data?

☒ Yes:
☐ No

If Yes: Describe how this software can be found and used.

The data is accessible and interpretable through open source software. We will describe the used software in the documentation.

9.6	Will a data repository be used for long term archiving and sharing of (meta)data?	☒ Yes:<br>☐ No
	If Yes: Which one(s)?	DANS EASY
9.7	Will an online catalogue or web portal be used to register the dataset?	☒ Yes:<br>☐ No
	If Yes: Which one(s)?	EU Clinical Trial Register and EudraCT/ToetsingOnline. It will also be automatically findable through DANS NARCIS.
9.8	Will a metadata schema be used to describe information about this project in the data repository?	☒ Yes:<br>☐ No
	If Yes: Describe which metadata schema(s) will be used.	This will be the Metadata scheme of the data repository. For DANS EASY this is Dublin Core.
9.9	Will a persistent identifier be assigned to the (meta)data in the online data repository to ensure persistent linking to the (meta)data?	☒ Yes:<br>☐ No
	If Yes: Which one(s)?	A DOI will be created through DataCite automatically when uploading to DANS EASY
9.10	Which data access strategy will be used?	☐ Open access<br>☐ Access for registered users<br>☒ Restricted access (on approval) → 9.12
9.11	Which license will be attached to the data?	
9.12	Are there any special conditions for sharing and reusing the data included in the terms of use?	☒ Yes:<br>☐ No
	If Yes: Describe the special conditions.	The data will be made available under an embargo 3 months.<br><br>A limited period of use will be included in the terms of use depending on the request.<br>The PI will verify the authenticity of the requesting researcher and will check whether their intentions are in line with the informed consent and whether the intended methodology is suitable, and will approve the request before providing access to the data.<br>Due to the sensitive nature of the data, collaboration is required.

		In order to prevent re-identification of the participants, combination with other data will be prohibited. A Data Transfer Agreement will be signed and the Technology Transfer Office will be involved to ensure the appropriate agreements are made.
9.13	Describe the process of how others can request access to the data and how the data will be made available to them.	Other researchers will be able to find the metadata in the data repository. They could express their interest in the dataset through emailing the PI. After meeting the sharing and reuse conditions as described above and approval of the PI, data access will be provided through the data repository.

#10 Costs

This section requires you to estimate the costs during and after the project.

10.1	Give an estimation of the total storage costs (€) and describe how these will be covered.	€0,- (not estimable, included in overhead)
10.2	Give an estimation of the total archive costs (€) and describe how these will be covered.	€1500,- covered by Spaarne Gasthuis
10.3	Give an estimation of the total data repository costs (€) and describe how these will be covered.	€0,-
10.4	Give an estimation of other data management related costs (€) and describe how these will be covered.	€0,-

The End
