# Supplementary material for: Safe, effective and cost-effective oxygen saturation targets for children and adolescents with respiratory distress: protocol for a randomised controlled trial (OxyKids study)
Source: BMJ Open. 2024 Dec 22;14(12):e087891. doi: 10.1136/bmjopen-2024-087891 (PMC11667372; doi:10.1136/bmjopen-2024-087891)
Supplement: online supplemental file 2 [file bmjopen-14-12-s002.pdf]

Subject information for participation in medical research

Safe and effective oxygen saturation targets for children with shortness of breath

Official title: Safe, effective and cost-effective oxygen saturation targets for children and adolescents with respiratory distress: a randomized controlled trial.

Introduction

Dear Sir/Madam,

With this letter, we would like to ask you and your child to take part in a medical study. Participation is voluntary. You have received this letter because you child has been admitted to the hospital with shortness of breath.
You can read about the medical study in this information sheet, what it means for you and your child, and what the pros and cons are. It is a lot of information. Can you please read the information and decide if you want to take part? If you want to take part, complete the form in Appendix C.

Ask your questions

You can make your decision based on the information in this information sheet. We also suggest that you do this:
- Ask the investigator who gave you this information your questions.
- Talk to your partner, family or friends about this study.
- Ask questions to the independent expert: Marieke Merelle, paediatrician in Spaarne Gasthuis. For contact details, go to appendix A.
- Read the information on www.rijksoverheid.nl/mensenonderzoek.

1. General information

This study has been set up by Spaarne Gasthuis, Amphia hospital in Breda, Isala in Zwolle, Sophia children's hospital in Rotterdam and the University Medical Centre in Groningen. The study is conducted by multiple paediatricians and multiple hospitals in the Netherlands participate. For this study, 560 children admitted to the hospital with shortness of breath are needed. The medical ethics review committee of Leiden Den Haag Delft has approved the study.

2. What is the purpose of the study?

The purpose of this study is to find out at which lower limit for saturation (amount of oxygen in the blood) we can best give extra oxygen to children that have been admitted for shortness of breath. We hope to accomplish a shorter period of illness for these children and that they can

be discharged home earlier. We compare a limit of 88% with an alternative limit of 92%. In other words, is it better to maintain a lower limit of 88% saturation or a lower limit of 92% in children admitted for shortness of breath.

3. What is the background of the study?

Children that have difficulty breathing often times have slightly less oxygen in their blood than healthy children have. That is why many children with shortness of breath in the Netherlands are given extra oxygen by nasal prongs or mask. It is how we make sure that organs receive enough oxygen. There are new insights that giving too much oxygen can be potentially harmful and that a little less oxygen in the blood does not hurt. Too much oxygen can for example lead to a slower recovery of shortness of breath. Also of importance is the fact that children using extra oxygen cannot be discharged home and thus have to prolong their stay in the hospital. We suspect that at present children will be supplied oxygen too readily and therefore remain unnecessary long in the hospital. Whether a child gets extra oxygen is determined by the amount of oxygen in the blood measured with an oximeter. An oximeter is a small strap or clip around the finger or toe that measures the amount of oxygen in the blood by means of a small light. The current lower limit to supply oxygen is 92%, but there is increasing evidence that this lower limit can be decreased. That would mean that currently children receive oxygen for too long of a period, resulting in an unnecessarily extended hospital stay and possibly also a longer period of being ill. However, this has not been sufficiently studied. Good research is important. If it is correct that less oxygen is needed then it may result in children being discharged home to recover sooner.. It is not relevant what the cause of the shortness of breath is.

4. What happens during the study?

How long will the study take?
Are you and your child taking part in the study? It will take 3 months in total after your child was discharged home. Your child will have been sufficiently recovered and he/she does not need supplemental oxygen. In those 3 months after discharge we would like for you (and your child) to fill out 3 questionnaires.

Step 1: are you eligible to take part?
First, we want to know if you are eligible to take part. That is the reason why we have set some criteria. The most important criteria are that your child has been admitted with shortness of breath, has a need for extra oxygen and no other serious disease. According to us your child fulfils these criteria and is eligible to take part in the study. Ask the doctor or researcher if you want to know exactly what the criteria to participate are.

If currently your child does not have a need for extra oxygen we still ask for your consent to participate in case extra oxygen is needed sometime during admission. In that way you do

not have to decide suddenly. In case your child does not need oxygen during admission we do not collect information on your child and you and your child are excluded from the study.

Step 2: the treatment

All children admitted with shortness of breath will be given an oximeter. This is part of standard care, thus also for children not participating in the study. The doctors and nurses agree on the lower threshold of oxygen saturation. We call this the saturation target. When your child's saturation is under the target we give extra oxygen. The usual target is 92%. We think that a lower target of 88% is better because we avoid giving unnecessary oxygen. If your child participates in the study a draw will decide whether we maintain a lower limit of 92% or 88%. You, the doctors or nurses cannot influence the draw.

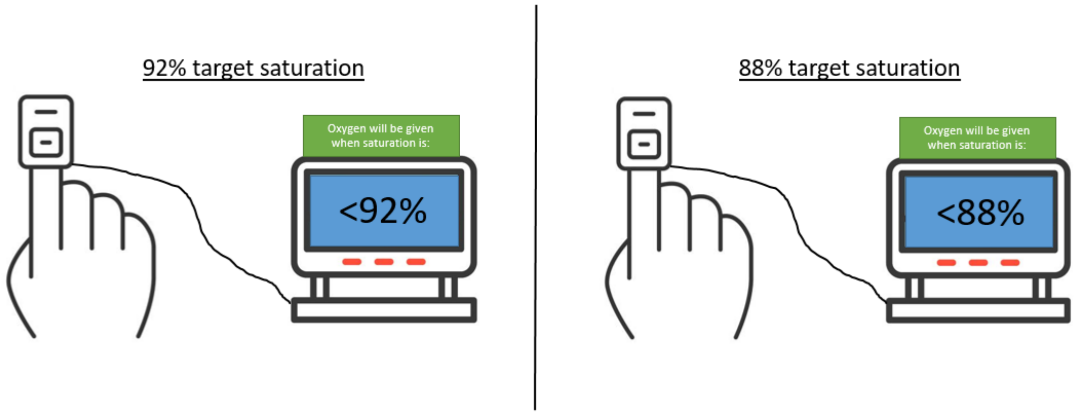

Figure 1: the two possible saturation targets

It is good to realize that doctors or nurses can start oxygen therapy for other reasons (for example if they want to try to make your child more comfortable). They are not prohibited from starting oxygen therapy if the saturation is above the target.

Sometimes children with shortness of breath need to be admitted to the intensive care unit because their condition worsens. That is mostly dependent on the level of shortness of breath. In case of admission to the intensive care unit a target saturation of 92% is maintained.

Step 3: study and measurements

Besides wanting to find out if children in the 88% will be discharged earlier we also want to know if the difference in saturation target is of influence on the duration of illness, the quality of life and activities of your child, experience of parents and costs incurred by parents/carers (such as missed work, travel expense) and the health care system. Health care costs are increasingly more important if we want to keep access to healthcare affordable for everyone. That is why we include questions about costs in this study. We follow you and your child three months from inclusion in the study. During that period we collect information from the medical chart of your child and we ask you (and possibly your child depending on his/her age) to fill out questionnaires upon discharge, and at 7, 28 and 90 days after discharge.

Filling out the questionnaires takes you a maximum of 30 minutes per questionnaire. You will receive an e-mail with a link to the questionnaire which you can complete from your computer, laptop or smartphone. The questionnaires are related to your child's health, how you have experienced the illness of your child and the incurred expenses. See the diagram below.

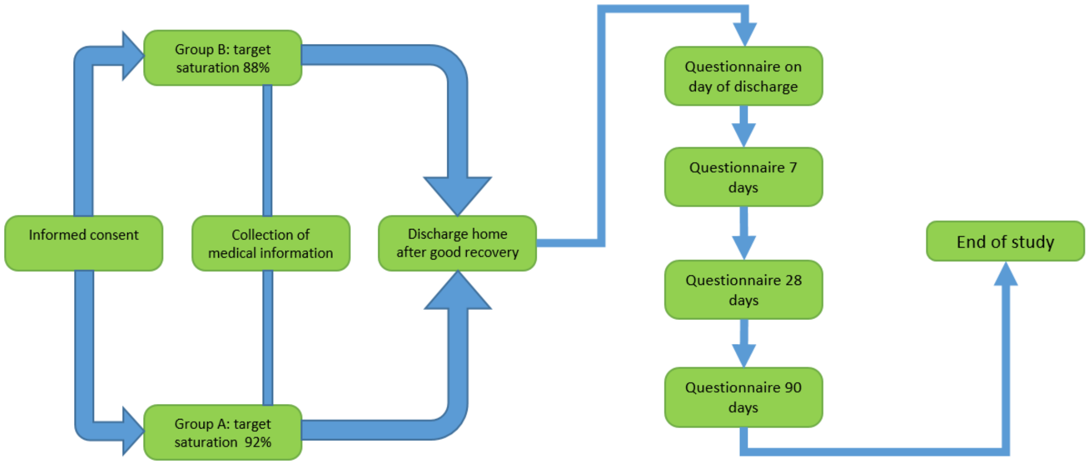

Figure 2: Study diagram, from enrollment to end of study

What is the difference with standard care?

This study is not very different from standard care other than that a different saturation target is maintained when your child is in the 88% group. The questionnaires during and after hospital admission are extra.

5. What agreements do we make with you?

We want the study to go well. That is why we want to make the following agreements with you:

- Your child does not take part in any other medical research during this study, it may influence our results.
- You fill out the questionnaires on the predetermined days.
- You should contact the investigator in these situations:
    - You child is being admitted to hospital care
    - You or your child no longer wants to take part in the study, this can occur at any moment without reason.
    - Your telephone number, address or email address changes.

6. What side effects, adverse effects or discomforts could you experience?

Oxygen therapy is part of the standard care you child receives. Regardless of whether your child participates in this study giving oxygen can lead to discomforts. It can cause irritation of the nasal mucosa which may result in a runny nose and crusts in the nose. Very rarely this

may lead to a nose bleed. Another discomfort will be that your child will be hampered in its movements since it will be attached to the oxygen tube.

7. What are the pros and cons if you take part in the study?

No possible negative side effects are expected by maintaining a lower saturation target of 88%. In previous studies a saturation target of 90% for children up to 12 months was safe and there are indications that that target can even be lower without negative health consequences. Still we will want to monitor if that is the case in our study. Therefore we will record whether the 88% group children will have to visit a doctor more frequent after discharge and if they resume their normal activities as quickly as their 92% counterparts.

Participating in this study also means spending extra time filling out the questionnaires.

Because a lower oxygen saturation is sometimes associated with brain damage we would like to mention this and make it clear why there is no such risk in this study. Children who develop less well due to lack of oxygen are children who were exposed to severe lack of oxygen (saturation far below 88%) or frequent desaturations during prolonged periods (months to years). In normal life even healthy bottle-fed babies appear to have saturations as low as 81%. By participating in this study it may happen that your child will have a lower saturation than usual for at most a few days, but always above 88%. Besides, the condition of your child is closely monitored by doctors and nurses, as with other children, and they can intervene when necessary. A risk of brain damage is out of the question.

You do not wish to participate in the study?
It is up to you to decide if you wish your child to participate in the study. Do you not wish to participate? Then your child will receive the standard treatment for shortness of breath and the current target saturation as stipulated by national guidelines will be maintained.

8. When does the study end?

In these situations, the study will stop for you:

- If your child had no need for supplemental oxygen during his/her hospital stay.
- All follow up questionnaires have been completed.
- You, or your child want(s) to stop participating in the study. You or your child can stop at any time. Report this to the investigator immediately. You do not have to explain why you or your child want(s) to stop, however this may help the investigator to improve the study or understand the results better. Your child will then get the standard treatment for his/her illness again.
- The investigator or doctor thinks it is better for you to stop.
- One of the following authorities decides that the study should stop:

- the government, or
- the Medical Ethics Review Committee assessing the study

What happens if you stop participating in the study?
The investigators use the data that have been collected up to the moment that you decide to stop participating in the study. The investigator may ask your permission for the use of some additional data. This is not compulsory.

The entire study ends when all the participants have finished.

The investigator will inform you about the most important results of the study after the study is completed. The whole study will take about 3,5 – 4 years, so it may take a while before you hear about the results.

9. Your child protests

It can happen that at one point during the study your child resists (does not cooperate). The investigator will have to stop the study immediately. It is difficult to describe what resistance is. An expression of resistance can differ with the age group your child is in. During this study your child will not be subjected to extra procedures that will be noticeable for your child. Before the start of the study we will discuss with you what will be perceived as resistance. The investigator will adhere to the code of conduct relating to expressions of objection by minors participating in medical research by the Netherlands Association for Pediatric research.

10. What will be done with your child's data

Is your child taking part in the study? Then you also give your consent to collect, use and store your and his/her data.

What data do we store?
Of your child
- name
- date of birth
- gender
- ethnicity
- information on your family composition
- information about your child's health
- (medical) information that we collect during the study
- skin type (can be of influence on the oxygen measurement accuracy)

Of you
- your name
- your age
- your address

- telephone number
- e-mail address
- information from the questionnaires that we collect during the study

Why do we collect, use and store your data?

We collect, use and store your data in order to answer the questions in this research. And to publish the results.

How do we protect your privacy?

To protect your privacy, we give a code to your and your child's data. We only put this code on your data. We keep the key to the code in a safe place in [local hospital]. When we process your data, we always use only that code. Even in reports and publications about the study, nobody will be able to see that it was about you or your child.

Who can see your data?

Some people can see your child's name and other personal information without a code. This could include data specifically collected for this study, but also data from the medical file. These are people checking whether the investigators are carrying out the study properly and reliably. These persons can access your data:

- Members of the committee that keeps an eye on the safety of the study.
- An auditor who is hired by the investigator/sponsor.
- National and international supervisory authorities.
- Members of the coordinating research team in the Spaarne Gasthuis can contact you by phone, if questionnaires have not been returned.

These people will keep your and your child's information confidential. We ask you to give permission for this access. The Health and Youth Inspectorate can access personal information without your permission.

For how long do we store your data?

We store your coded data in the research centre for 25 years.

Can we use your data and body material for other research?

Your and your child's collected data may also be important for other medical research in the area of children with shortness of breath. For this purpose, your child's data remains in the research centre for 25 years. Please indicate in the consent form whether you agree with this. Do you not want to give your consent? Then your child can still take part in this study. You will get the same healthcare.

Can you take back your consent for the use of your data?

You can take back your consent for the use of your and your child's data at any time. Please tell the investigator if you wish to do so. This applies both to the use in this study and to the use in other medical research. But please note: if you take back your consent, and the

investigators have already collected data for research, they are still allowed to use this information.

Do you want to know more about your privacy?

- Do you want to know more about your rights when processing personal data? Visit https://www.autoriteitpersoonsgegevens.nl/en.
- Do you have questions about your rights? Or do you have a complaint about the processing of your personal data? Please contact the person who is responsible for processing your personal data. For the present study, this is:
    - See Appendix A for contact details, and website.
- If you have any complaints about the processing of your personal data, we recommend that you first discuss them with the research team. You can also contact the Data Protection Officer of [the institution]. Or you can submit a complaint to the Dutch Data Protection Authority.

Where can you find more information about the study?
You can find more information about the study on the following website:
https://zorgevaluatienederland.nl/evaluations/oxykids

11. What happens after the study?

Will you receive the results of the study?
About one year after the study is completed the investigator will let you know what the most important results of the study are.

12. Will you or your child receive compensation for participation in the study?

Participation of your child in the study will not cost you anything. Neither you or your child will get any compensation when taking part in this study.

13. Are you insured during the study?

Insurance has been taken out for everyone who takes part in this study. The insurance pays for damage caused by the study. But not for all damage. You can find more information about this insurance and any exceptions in **Appendix B**. It also says who you can report damage to.

14. We will inform your general practitioner

The treating physician will let your general practitioner know in the discharge letter that your child participates in this study. This is for your child's safety, so that your general practitioner is informed as well.

15. Do you have any questions?

You can ask questions about the study to the investigator. Would you like to get advice from someone who is independent from the study? Then contact the independent expert: Marieke Merelle. She knows a lot about the study, but is not a part of this study. Contact details can be found in Appendix A.
Do you have a complaint? Discuss it with the investigator or the doctor who is treating you. If you prefer not to do so, please visit the complaints officer/complaints committee of your hospital/institute. **Appendix A** tells you where to find this.

16. How do you give consent for the study?

You can first think carefully about this study. Then you tell the investigator if you understand the information and if you want to take part or not. If you want to take part, fill in the consent form that you can find with this information sheet. You and the investigator will both get a signed version of this consent form.
Thank you for your attention.

Appendices to this information

A. Contact details
B. Information about the insurance
C. Consent form(s)

Appendix A: contact details for [name of participating centre]

Investigator
S. Louman, arts-onderzoeker
E-mail: oxykids@spaarnegasthuis.nl
By phone: 023 – 2241645 office hours

Study nurse
[name]
E-mail:
By phone:

Independent expert:
Paediatrician [redacted]
E-mail: [redacted]
By phone: [redacted] office hours

Complaints:
Klachtencommissie **[name of participating centre]**By phone:

Data Protection Officer of **[name of participating centre]**:
E-mail:
Dept.: Juridische zaken
By phone:

Appendix B: information about the insurance

Spaarne Gasthuis has taken out insurance for everyone who takes part in the study. The insurance pays for the damage you have suffered because you participated in the study. This concerns damage you suffer during the study or within 4 years after you participated in the study. You must report damage to the insurer within 4 years.

Has your child suffered damage as a result of the study? Please report this to this insurer:

The insurer of the study is:	
Name insurer:	Centramed B.A.
Adres:	Maria Montessorilaan 9, 2719 DB, Zoetermeer
Telefoonnummer:	070-3017070
E-mail:	info@centramed.nl
Policy number:	624.100.033

The insurance pays a maximum of €650.000 per person and €5,000,000 for the entire study and € 7,500,000 per year for all studies by the same sponsor.

Please note that the insurance does **not** cover the following damage:

- Damage due to a risk about which we have given you information in this sheet. But this does not apply if the risk turned out to be greater than we previously thought. Or if the risk was very unlikely.
- Damage to your child’s health that would also have happened if you had not taken part in the study.
- Damage that happens because you did not follow directions or instructions or did not follow them properly.
- Damage caused by a treatment method that already exists. Or by research into a treatment method that already exists.

These provisions can be found in the 'Besluit verplichte verzekering bij medisch-wetenschappelijk onderzoek met mensen 2015' ('Medical Research (Human Subjects) Compulsory Insurance Decree 2015'). This decision can be found in the Government Law Gazette (https://wetten.overheid.nl).

Appendix C: Informed consent form - Parent , carer or guardian

Belonging to

Effective and safe oxygen saturation for children with shortness of breath

I have been asked to give consent for my child to participate in this medical study:

Name of subject (child): ……………………………..Date of birth: __/__/__

- I have read the information sheet for subjects/parents/guardians. I was able to ask questions. My questions have been answered well enough. I had enough time to decide if I want my child to take part.
- I know that taking part is voluntary. I also know that I can decide at any time that my child will not take part after all. I do not have to explain why.
- I give consent to inform my general practitioner that my child is taking part in this study.
- I give consent to collect and use my child's data. The investigators only do this to answer the question of this study.
- I know that some people will be able to see all of my child's data to review the study. These people are mentioned in this information sheet. I give consent to let these people see my child's data for this review.
- Please tick yes or no in the table below.
- I give consent that a member of the central research team of Spaarne Gasthuis can view contact details in order to call me if necessary. This is also explained in this information letter.

I give consent to have my child's data stored for use in other research, as stated in the information sheet.	Yes ☐	No ☐
I give consent to ask my child after this study if he/she wants to participate in a follow-up study.	Yes ☐	No ☐

- I agree that my child takes part in this study.

Email address to which the questionnaire can be send:
|__|__|__|__|__|__|__|__|__|__|__|__|__|__|__|__|__|__|__|__|__|__|__|__|__|__|__|__|__|__|__

Most commonly used phone number parents/guardians:
|__|__|__|__|__|__|__|__|__|__|__|__|

Parent/guardian name**: ………………………………

Signature: ………………………………… Date: __/__/__

Other parent/guardian name**: …………………….

Signature: ……………………………………… Date: __/__/__

I declare that I have fully informed the person(s) mentioned above about the said study.

If any information becomes known during the study that could influence the parent/guardian's consent, I will let them know in good time.

Investigator name (or their representative): …………………………

Signature: ……………………… Date: __/__/__

The parent/guardian will receive a complete information sheet, together with a signed version of the consent form.
