# Supplementary material for: Safe, effective and cost-effective oxygen saturation targets for children and adolescents with respiratory distress: protocol for a randomised controlled trial (OxyKids study)
Source: BMJ Open. 2024 Dec 22;14(12):e087891. doi: 10.1136/bmjopen-2024-087891 (PMC11667372; doi:10.1136/bmjopen-2024-087891)
Supplement: online supplemental file 3 [file bmjopen-14-12-s003.pdf]

The OxyKids Study

Data Safety and Monitoring Board Charter

Version	1.3
Date	03-06-2023
Study title	Safe, effective and cost-effective saturation targets for children with respiratory distress: a randomized controlled trial.
Short title	OxyKids Study
Protocol ID	2022.0100
Registration	To be determined
Funding	ZonMW Doelmatigheid nr. 10390012110075 en Stichting Astma Bestrijding
Principal Investigator	Dr. A. L. M. Boehmer, MD PhD<br>Pediatric Pulmonologist<br>Spaarne Gasthuis<br>Spaarnepoort 1, 2134 TM, Hoofddorp<br>Phone: +3120 – 2245760<br>E-mail: aboehmer@spaarnegasthuis.nl

I. Outline of scope of charter

The purpose of this document is to describe the roles and responsibilities of the independent Data Safety and Monitoring Board (DSMB) for the OxyKids study, including the timing of meetings, methods of providing information to and from the DSMB, frequency and format of meetings, statistical issues and relationships with other committees.

II. Introduction to the OxyKids study

Rationale: Hypoxemia as measured with pulse oximetry is one of the most frequent reasons for hospital admission in children with respiratory distress. However, the lower limits for supplemental oxygen administration in children with respiratory diseases are poorly studied. Current guidelines recommend treatment with supplemental oxygen if oxygen saturation (SpO2) falls below 90-94%, but these recommendations are based on low levels of evidence. As the duration of hospital stay likely depends on duration of oxygen supplementation, lower SpO2 limits to start or discontinue supplemental oxygen, may reduce length of hospital stay and negative effects of prolonged hospitalization. Therefore, the aim of this study is to determine adequate (ie. safe and effective) lower SpO2 thresholds for children admitted to hospital with respiratory diseases.

Objective: Primary objective: to investigate if an SpO2 threshold of 88% for children and adolescents with respiratory distress results in a safe reduction of length of hospital stay, compared to an SpO2 threshold of 92%.

Secondary objectives: to investigate if a lower SpO2 threshold of 88% leads to differences in length and severity of symptoms, readmissions, time to normal activity, patient and parent quality of life and cost-effectiveness compared to an SpO2 threshold of 92%.

Study design: multicenter open-label randomized controlled trial with cost-effectiveness analysis.

Study population: Children aged 6 weeks to 12 years, hospitalized with lower respiratory disease due to bronchiolitis, viral wheeze or lower respiratory tract infection, as diagnosed by the treating physician, requiring supplemental oxygen (SpO2 < 92% or for treating symptoms of respiratory distress). Exclusion criteria: children with other preexisting respiratory diseases, cardiovascular, neurological or hematological conditions and children born <32 weeks gestational age, or previously included in the study.

Intervention: Participants in the intervention group will have an SpO2 threshold of 88% for starting and stopping supplemental oxygen.

Main study parameters/endpoints: Primary outcome is the time from admission to meeting all discharge criteria. Secondary outcomes include length of stay, duration of illness, readmissions/reassessments, and quality of life. A cost-effectiveness analysis will be performed alongside.

Patients are followed up to 90 days after inclusion, with parents / caregivers and/or children answering a digital questionnaire at discharge and after 7, 28 and 90 days.

III. Roles and Responsibilities

Aims of the DSMB

The aims of the DSMB are to protect and serve the study participants regarding safety, to monitor the overall conduct of the clinical study and to assist and advise the Principal Investigators so as to protect the validity and credibility of the trial.

Terms of reference

The DSMB should receive and review the progress and accruing data of this trial and provide advice on the conduct of the trial to the Trial Steering Committee. The DSMB should inform the Principle Investigator if, in their view:

(i) the results are likely to convince a broad range of clinicians, including those supporting the trial and the general clinical community, that on balance one trial arm is clearly contra-indicated for all participants or a particular category of participants, and there was a reasonable expectation that this new evidence would materially influence patient management (harm); or

(ii) the DSMB should perform interim review of the trial's progress including updated figures on recruitment, data quality, and safety data.

Specific roles of the DSMB

- monitor recruitment figures and losses to follow-up
- monitor evidence for treatment harm (e.g. SAEs, duration of symptoms, hospital readmissions/reassessments, number of PICU admissions)

- decide whether to recommend that the study continues to recruit participants or whether
- recruitment should be terminated either for everyone or for some treatment groups and/or some participant subgroups
- suggest additional data analyses
- advise on protocol modifications suggested by investigators or sponsors (e.g. to inclusion criteria, study endpoints, or sample size)
- monitor planned sample size assumptions
- monitor compliance with previous DSMB recommendations
- considering the ethical implications of any recommendations made by the DSMB
- assess the impact and relevance of external evidence

IV. Before or early in the trial

All potential members should have sight of the protocol before agreeing to join the DSMB. Before recruitment begins the study has undergone review by the funder, sponsor and the MERC LDD. Therefore, if a potential DSMB member has major reservations about the study (e.g. the protocol or the logistics) they should report these to the trial office and may decide not to accept the invitation to join.

DSMB members should be independent and constructively critical of the ongoing trial, but also supportive of aims and methods of the trial.

The DSMB meets before the trial starts or early in the course of the trial, to discuss the protocol, the trial, any analysis plan, future meetings, and to have the opportunity to clarify any aspects with the principal investigators. The DSMB should meet within one year of recruitment commencing.

V. Composition of the DSMB

The members of the DSMB are independent of the trial and any competing interests, both real and potential will be declared. A short competing interest form should be completed and returned by the DSMB members to the trial coordinating centre (Appendix 1).

The members of the DSMB of the OxyKids study are:

- Member 1: Dr. Peter Merkus, Pediatric pulmonologist at Radboud Medical Centre Nijmegen
- Member 2: Judith Vonk, Pulmonary Epidemiologist at University Medical Centre Groningen
- Member 3: Dr. Gavin W. ten Tusscher, pediatrician at Dijklander Ziekenhuis Hoorn

Given her previous experience of serving on DSMBs, Judith Vonk will be the chair of this DSMB. The Chair is capable to facilitate and summarise discussions.

The trial statistician (E. R. Andrinopoulou) will produce (or oversee the production of) the report to the DSMB and will participate in DSMB meetings, guiding the DSMB through the report, participating in DSMB discussions and, on some occasions, taking notes.
The project leader/principal investigator, may be asked, and should be available, to attend open sessions of the DSMB meeting. The other Trial Management Group members will not usually be expected to attend but can attend open sessions when necessary.
The DSMB has an advisory role (makes recommendations), not an executive role (makes decisions).
DSMB members will only be reimbursed for travel and accommodation costs.

VI. Organization of DSMB meetings

The DSMB will meet to discuss the findings of the interim/safety analyses. These will be conducted when 25% of the data has been gathered (i.e. after 140 patients have completed follow-up).
Meetings will be face-to-face if possible, with teleconference as a second option.

Effort should be made for all members to attend. The trial manager will try to ensure that a date is chosen to enable this. Members who cannot attend in person should be encouraged to attend by teleconference. If a member does not attend a meeting, it should be ensured that the member is available for the next meeting. If a member does not attend a second meeting, they should be asked if they wish to remain part of the DSMB. If a member does not attend a third meeting, they should be replaced. Meetings will consist of an open and a closed part. During closed meetings only the DSMB members are present and others whom they specifically invite, e.g. the trial statistician (E.R. Andrinopoulou). In open sessions, all those attending the closed session are joined by the project leader/principal investigator (dr. A. L. M. Boehmer), the trial manager/clinical trial coordinator (drs. S. Louman) and representatives of the sponsor or regulator, as relevant.

VII. Trial documentation and procedures to ensure confidentiality and proper communication

Intended content of material to be available in open sessions: Accumulating information relating to recruitment and data quality (e.g. data return rates, treatment compliance) will be presented. Total numbers of events for the primary outcome measure and other outcome measures may be presented, at the discretion of the DSMB.
Intended content of material to be available in closed sessions: In addition to all the material available in the open session, the closed session material will include safety data by treatment group. The DSMB will be blinded to the treatment allocation. The chair of the DSMB will have a sealed envelope containing the randomization codes, to reveal the allocation labels if the DSMB thinks this is necessary. The accumulating data and interim analysis will only be available to the DSMB. DSMB members do not have the right to share confidential information with anyone outside the DSMB, including the project leader/principal investigator. Identification and circulation of external evidence (e.g. from other trials/ systematic reviews) is not the responsibility of the DSMB members. The project leader/principal investigator or the trials office team will usually collate any such information.
The DSMB reports its recommendations in writing to the Trial Steering Committee. This will be copied to the trial statistician. If the trial is to continue largely unchanged then it is often useful for the report from the DSMB to include a summary paragraph suitable for study promotion purposes.
The DSMB will receive relevant reports at least 2 weeks before any meetings. If preferred papers will be brought to face-to-face meetings by the trial statistician; time would then be needed for DSMB members to assimilate the report. The DSMB members should store the papers safely after each meeting so they may check the next report against them. After the trial is reported, the DSMB members should destroy all interim reports.

VIII. Decision making

Safety analyses will be performed when approximately 25% of the patients have reached the end of the follow-up. Safety outcomes will be analyzed (duration of symptoms, reattendence to health care, PICU admissions), as well as SAEs, using the statistical guidelines as decided at the pre-recruitment DSMB meeting.

The possible recommendations that will be open to the DSMB are:

- No action needed, trial continues as planned
- Early stopping due, for example, to clear harm of a treatment, or external evidence. In principle, the trial will not be stopped early for beneficial effect of the intervention on the primary outcome before the minimum number of evaluable patients required (560) are included. Hence, there will be no alpha spending associated with the interim analyses.
- Stopping recruitment within a subgroup.
- Extending recruitment (based on actual control arm response rates being different to predicted rather than on emerging differences) or extending follow-up.
- Sanctioning and/or proposing protocol changes

Every effort should be made for the DSMB to reach an unanimous decision. If the DSMB cannot achieve this, a vote may be taken, although details of the vote should not be routinely included in the report to the Trial Steering Committee as these may inappropriately convey information about the state of the trial data. It is important that the implications (e.g. ethical, statistical, practical, financial) for the trial be considered before any recommendation is made.

If the report is circulated before the meeting, DSMB members who will not be able to attend the meeting may pass comments to the DSMB Chair for consideration during the discussions. If the DSMB is considering recommending major action during meeting where not all members could attend in person or by teleconference, the DSMB Chair should talk with the absent members as soon after the meeting as possible to check they agree. If they do not, a further teleconference should be arranged with the full DSMB.

IX. Reporting

The DSMB will report their recommendations and decisions in a (digital) letter to the Trial Steering Committee (A. Boehmer, M. Pijnenburg, G. Koppleman, S. Louman) within three weeks after the DSMB meeting. Separate minutes will be made by the secretary (one of the DSMB members) assigned by the Chair. The DSMB Chair should sign off any minutes or notes. Minutes of the closed session will be kept by the secretary and the Chair.

If the DSMB has serious problems or concerns with the Trial Steering Committee decision a meeting of these groups should be held. The information to be shown would depend upon the action proposed and the DSMB's concerns. Depending on the reason for the disagreement, confidential data will often have to be revealed to all those attending such a meeting. The meeting should be chaired by an external expert who is not directly involved with the trial. Should the sponsor decide not to fully implement the advice of the DSMB, the sponsor will send the advice to the reviewing METC, including a note to substantiate why (part of) the advice of the DSMB will not be followed.

X. After the trial

At the end of the trial there will be a meeting to allow the DSMB to discuss the final data with

principal trial investigators and give advice about data interpretation. The DSMB may wish to see a statement that the trial results will be published in a correct and timely manner.
DSMB members will be named and their affiliations listed in the main report, unless they explicitly request otherwise. A brief summary of the timings and conclusions of DSMB meetings will be included in the body of this paper.
The DSMB may wish to be given the opportunity to read and comment on any publications before submission. The DSMB may discuss issues from their involvement in the trial 12 months after the primary trial results have been published or earlier if the Steering Committee agrees.

Competing interests form

Potential competing interests of Data Monitoring Safety Board members for the OxyKids Study (2022.0100)

The avoidance of any perception that members of a DSMB may be biased in some fashion is important for the credibility of the decisions made by the DSMB and for the integrity of the trial. Possible competing interest should be disclosed via the trials office. In many cases simple disclosure up front should be sufficient. Otherwise, the (potential) DSMB member should remove the conflict or stop participating in the DSMB. Table 1 lists potential competing interests.

Table 1 - Potential competing interests

• Stock ownership in any commercial companies involved
• Stock transaction in any commercial company involved (if previously holding stock)
• Consulting arrangements with the sponsor
• Frequent speaking engagements on behalf of the intervention
• Career tied up in a product or technique assessed by trial
• Hands-on participation in the trial
• Involvement in the running of the trial
• Emotional involvement in the trial
• Intellectual conflict e.g. strong prior belief in the trial's experimental arm
• Involvement in regulatory issues relevant to the trial procedures
• Investment (financial or intellectual) in competing products
• Involvement in the publication

Please complete the following section and return to the trials office.
Please provide details of any competing interests:

Date: 31 january 2023

Name: Judith M. Vonk

Signature: [redacted]

X **No,** I have no competing interests to declare
☐ **Yes,** I have competing interests to declare (please detail below)

APPENDIX 1.

Competing interests form

Potential competing interests of Data Monitoring Safety Board members for the OxyKids Study (2022.0100)

The avoidance of any perception that members of a DSMB may be biased in some fashion is important for the credibility of the decisions made by the DSMB and for the integrity of the trial. Possible competing interest should be disclosed via the trials office. In many cases simple disclosure up front should be sufficient. Otherwise, the (potential) DSMB member should remove the conflict or stop participating in the DSMB. Table 1 lists potential competing interests.

Table 1 - Potential competing interests

• Stock ownership in any commercial companies involved
• Stock transaction in any commercial company involved (if previously holding stock)
• Consulting arrangements with the sponsor
• Frequent speaking engagements on behalf of the intervention
• Career tied up in a product or technique assessed by trial
• Hands-on participation in the trial
• Involvement in the running of the trial
• Emotional involvement in the trial
• Intellectual conflict e.g. strong prior belief in the trial's experimental arm
• Involvement in regulatory issues relevant to the trial procedures
• Investment (financial or intellectual) in competing products
• Involvement in the publication

Please complete the following section and return to the trials office.
Please provide details of any competing interests:

Date: april 21st 2023

Name: Signature: [redacted]

Peter JFM Merkus, MD PhD, associate professor

XX ☐ **N o,** I have no competing interests to declare

[redacted]

APPENDIX 1.

Competing interests form

Potential competing interests of Data Monitoring Safety Board members for the OxyKids Study (2022.0100)

The avoidance of any perception that members of a DSMB may be biased in some fashion is important for the credibility of the decisions made by the DSMB and for the integrity of the trial. Possible competing interest should be disclosed via the trials office. In many cases simple disclosure up front should be sufficient. Otherwise, the (potential) DSMB member should remove the conflict or stop participating in the DSMB. Table 1 lists potential competing interests.

Table 1 - Potential competing interests

• Stock ownership in any commercial companies involved
• Stock transaction in any commercial company involved (if previously holding stock)
• Consulting arrangements with the sponsor
• Frequent speaking engagements on behalf of the intervention
• Career tied up in a product or technique assessed by trial
• Hands-on participation in the trial
• Involvement in the running of the trial
• Emotional involvement in the trial
• Intellectual conflict e.g. strong prior belief in the trial's experimental arm
• Involvement in regulatory issues relevant to the trial procedures
• Investment (financial or intellectual) in competing products
• Involvement in the publication

Please complete the following section and return to the trials office.
Please provide details of any competing interests:

Date: 07-06-2023

Name: GW ten Tusscher

Signa[redacted]

☒ **No,** I have no competing interests to declare
☐ **Yes,** I have competing interests to declare
