# Supplementary material for: Safe, effective and cost-effective oxygen saturation targets for children and adolescents with respiratory distress: protocol for a randomised controlled trial (OxyKids study)
Source: BMJ Open. 2024 Dec 22;14(12):e087891. doi: 10.1136/bmjopen-2024-087891 (PMC11667372; doi:10.1136/bmjopen-2024-087891)
Supplement: Supplementary file 4 [file bmjopen-2024-087891.reviewer_comments.pdf]

PEER REVIEW HISTORY

BMJ Open publishes all reviews undertaken for accepted manuscripts. Reviewers are asked to complete a checklist review form and are provided with free text boxes to elaborate on their assessment. These free text comments are reproduced below.

ARTICLE DETAILS

Title (Provisional)

Safe, Effective and Cost-Effective Oxygen Saturation Targets for Children and Adolescents with Respiratory Distress: Protocol for a Randomized Controlled Trial (OxyKids Study)

Authors

Louman, Sam; van Stralen, Karlijn J.; Koppelman, Gerard; Vaessen-Verberne, Anja; Bekhof, Jolita; Bosmans, J; Brackel, Caroline; Kamps, Arvid W. A.; de Kleer, Ismé; Balemans, Walter; Scheffer, Mirjam; Brouwer, Marianne; van den Beukel, Marije; Andrinopoulou, Eleni-Rosalina; Pijnenburg, Mariëlle W. H.; Boehmer, Annemie L.M.

VERSION 1 - REVIEW

**Reviewer**	**1**
**Name**	**Schuh, Suzanne**
**Affiliation**	**The Hospital for Sick Children, pediatrics**
**Date**	**30-Jun-2024**
**COI**	**None**

This is a well-justified multi-centre open-label parallel group trial of children 6 weeks -12 years of age hospitalized for respiratory distress due to physician-diagnosed bronchiolitis, viral wheeze or a lower-respiratory tract infection and requiring supplemental oxygen as per saturation<92%/clinical indication. These children will be randomized to target oxygen saturations of 88% vs 92% to determine if there is a significant group difference in the time to predetermined dischargeability criteria. The secondary objectives are to examine the group differences in the length of hospital stay, time on oxygen, various safety outcomes, re-visits, parental anxiety, quality of life and cost effectiveness from the societal perspective.

The authors are to be commended for undertaking this challenging task, which, when done well, can produce useful and practice-changing results. With this goal in mind, there are several points which warrant consideration in the Methods section:

1- Lack of blinding: this is indeed a major limitation posing potential introduction of ascertainment bias, and a reference specifically prohibiting oximeter data alteration would be important. Because the measurement error of most saturation monitors is about 2%,

manipulation of the saturation values by this approx amount would be ideal. The authors should specify where this blinding block arises from (the E.U.vs other). Because you cannot blind the study, desaturations probably represent the most objective justification for implementing oxygen therapy, more so than physician judgment or parental request. It therefore seems prudent to document why oxygen therapy was commenced.

2- Further description of "lower respiratory infection" would be helpful: for example, will the study patients require chest radiograph/point-of-care-ultrasound for confirmation of this diagnosis? The same would be true of criteria for bronchiolitis (age cut-off, 1st vs recurrent episode etc).

3- While I understand that the criteria for starting supplemental oxygen therapy should be pragmatic, special attention needs to be paid to the quality of the waveform on the monitor and the clinical status of the patient. Importantly, young children often desaturate during sleep without any clinical decompensation, with comparable outcomes to those who do not desaturate (Principi et al, JAMA Pediatr 2016), so limiting the saturation measurements to those measured during awake state would be critical. This is also important for the primary outcome of the study.

4- One of the secondary outcomes are the re-visits. When will this be measured?

5- Feasibility: I cannot find this section in the protocol. It will determine the study duration and needs estimates on the number of patients needed to be screened, expected to be eligible, the refusal rate, loss-to-follow-up and the number of children available for the primary analysis. Where does the 10% loss to follow up come from?

6- A detailed description of when, how often and by whom the discharge criteria will be assessed is important as this is the primary outcome.

7- Good luck and I look forward to the fruits of your work!

Reviewer 2

Name Caino de Oliveira , Felipe Rezende

Affiliation Santa Catarina Hospital, Department of Pediatrics, Brazil; Alvorada Hospital, Pediatric Intensive Care Unit, Brazil; GRAACC/IOP, Pediatric Intensive Care Unit, Brazil; Department of Pediatrics,

Date 10-Jul-2024

COI no conflicts

After analysis, well described and aligned for collection

VERSION 1 - AUTHOR RESPONSE

Reviewer: 1

Comments to the Author:

This is a well-justified multi-centre open-label parallel group trial of children 6 weeks -12 years of age hospitalized for respiratory distress due to physician-diagnosed bronchiolitis, viral wheeze or a lower-respiratory tract infection and requiring supplemental oxygen as per saturation<92%/clinical indication. These children will be randomized to target oxygen saturations of 88% vs 92% to determine if there is a significant group difference in the time to predetermined dischargeability criteria. The secondary objectives are to examine the group differences in the length of hospital stay, time on oxygen, various safety outcomes, re-visits, parental anxiety, quality of life and cost effectiveness from the societal perspective.
The authors are to be commended for undertaking this challenging task, which, when done well, can produce useful and practice-changing results. With this goal in mind, there are several points which warrant consideration in the Methods section:

Response: we thank the reviewer for the insightful comments which have helped us improve our manuscript and has given us food for thought for any future publications. Below we provide a point-by-point response of the changes made to the manuscript.

1- Lack of blinding: this is indeed a major limitation posing potential introduction of ascertainment bias, and a reference specifically prohibiting oximeter data alteration would be important. Because the measurement error of most saturation monitors is about 2%, manipulation of the saturation values by this approx amount would be ideal. The authors should specify where this blinding block arises from (the E.U. vs other). Because you cannot blind the study, desaturations probably represent the most objective justification for implementing oxygen therapy, more so than physician judgment or parental request. It therefore seems prudent to document why oxygen therapy was commenced.

Response: we fully agree that lack of blinding is a major limitation. We know that in the BIDS study manipulation with oximeters with the help of the manufacturer was used for double blinding (Cunningham S. et al Lancet 2015 Sept.). To our regret, the 2 main manufacturers of oximeter devices, with who's scientific and management board we have had good discussions, no longer allow manipulation of those devices. We designed this study with manipulated oximeters and contacted Masimo. At first they were interested in helping with the study, but when EU CE regulation changed in 2022 they became more strict. They told us: "The [international research] committee [of Masimo] identified that the methodology is not consistent with [manufacturer]-recommended best practices due to the need for manipulation of the oximeter device. [Manufacturer] policy does not allow this practice per company procedure." The second largest manufacturer gave the same answer when asked for their support. As the reviewer suggests, we have specified this in the manuscript:

Line 217-220: "Although altering devices as done in the study of Cunningham et al [8] would be ideal, device manufacturers are no longer willing to allow for such modifications due to tightened EU medical Devices Regulation requirements and liability concerns."

We agree with the reviewer that it is important to document the reasons for starting oxygen therapy. In our trial, we document whether oxygen therapy was initiated because of hypoxemia or for clinical reasons such as respiratory rate, heart rate and work of breathing. A fixed set of clinical parameters at start of oxygen therapy are also recorded. This is specified in lines 203-205: "Study protocol adherence is monitored by the registration of start and stop times of supplemental oxygen administration, including the reasons for starting and stopping, and clinical symptoms of the patient such as respiratory rate, heart rate and work of breathing."

Lack of blinding and the necessity for documenting reasons for starting and stopping oxygen therapy has also been included in the new Discussion paragraph, which we decided to add to the manuscript to address these and other discussion points raised by the reviewer. The relevant section in the discussion paragraph reads as follows at line 525-530: "The open-label design of the trial introduces a risk of bias that is difficult to overcome without manipulating pulse oximeter equipment, a procedure that manufacturers did not want to facilitate. To mitigate this risk all study personnel, clinicians and nurses undergo thorough training to follow the study procedures. Additionally, to quantify any potential bias, clinical parameters such as respiratory rate, heart rate and work of breathing at start and stop of supplemental oxygen will be recorded."

2- Further description of "lower respiratory infection" would be helpful: for example, will the study patients require chest radiograph/point-of-care-ultrasound for confirmation of this diagnosis? The same would be true of criteria for bronchiolitis (age cut-off, 1st vs recurrent episode etc).

Response: we agree and have clarified how diagnoses are defined. Point of care ultrasound or chest radiograph are not standard of care to diagnose lower respiratory tract infection in The Netherlands and as such are not required in the study. See line 156-162: "As a pragmatic approach, diagnoses are determined by the treating physician. They are free to use diagnostic tools and/or criteria as per usual care. The diagnosis of lower respiratory tract infection is any lower respiratory tract infection other than bronchiolitis. This can be a clinical diagnosis, not necessarily confirmed by chest radiograph or point of care ultrasound. In the case of overlapping diagnoses, the most prominent diagnosis is registered. If the diagnosis is changed during admittance, the diagnosis at enrolment will be used."

3- While I understand that the criteria for starting supplemental oxygen therapy should be pragmatic, special attention needs to be paid to the quality of the waveform on the monitor and the clinical status of the patient. Importantly, young children often desaturate during sleep without any clinical decompensation, with comparable outcomes to those who do not desaturate (Principi et al, JAMA Pediatr 2016), so limiting the saturation measurements to those measured during awake state would be critical. This is also important for the primary outcome of the study.

Response: We thank the reviewer for the insightful feedback regarding oxygen saturation monitoring in young children. As Principi et al (JAMA Pediatr 2016) show, desaturations can occur in sleep without clinical deterioration in young children with bronchiolitis. As the reviewer states, and which is corroborated by further evidence (McCulloh et al, Jama Pediatr 2015), it is likely that continuous saturation monitoring especially during sleep is likely to unnecessarily escalate treatment and prolong hospitalization in infants with bronchiolitis. In our study, we aim to compare two oxygen saturation thresholds within the current standard of care (continuous saturation monitoring). Therefore, limiting saturation measurements to during awake state is not in line with current standards of care and not the aim of our RCT. In training nursing staff however, we do set guidelines for determining the need for supplemental oxygen. Nurses are instructed to wait up to 15 minutes during a desaturation and monitor work of breathing, before giving supplemental oxygen. Discharge criteria include maintaining saturations above the threshold for at least 4 hours, which must include a sleep period for patients under 2 years. While standard practice may lag behind available evidence, altering it extensively within one trial could affect the trial's external validity.

We have addressed this in the new Discussion paragraph, the relevant section can be found at line 531-539: "Due to the pragmatic design, children in the OxyKids trial will have continuous oxygen saturation monitoring. As was shown previously, hypoxemia can occur in sleep without clinical deterioration in young children with bronchiolitis [29]. Moreover, continuous oxygen saturation monitoring may unnecessarily escalate treatment and prolong hospitalization in infants

with bronchiolitis [30]. However, as the OxyKids trial compares different saturation thresholds within the current standard of care in both children with bronchiolitis and older children with other diagnoses, limiting oxygen saturation measurements only to awake state is not in line with the aim of the trial, even though doing so could be the better clinical practice."

4- One of the secondary outcomes are the re-visits. When will this be measured?

Response: these are measured at 28 days. Parents report readmissions or visits to health care providers in a digital questionnaire. Readmissions within 28 days likely to be related to the study intervention are also reported as SAE's by the participating centres. We have specified this in the manuscript. See lines 252-254 under 'Safety outcomes': "Number of unscheduled readmissions and revisits to healthcare providers, as reported by parents in follow-up questionnaires and registered from patient electronic health records." And see under SAE that require reporting at lines 469-470: "other SAEs likely related to the study intervention, such as readmissions within 28 days."

5- Feasibility: I cannot find this section in the protocol. It will determine the study duration and needs estimates on the number of patients needed to be screened, expected to be eligible, the refusal rate, loss-to-follow-up and the number of children available for the primary analysis. Where does the 10% loss to follow up come from?

Response: the section has been added under 'Sample size and feasibility' at lines 308-312: "This is feasible, as two participating centres admit an average of 200 children aged 0-12 years with respiratory disease per year. Estimating 80% eligibility, 15% not approached, and 60% willingness to participate (based on parent interviews, in-hospital data and expert estimation), and with 10 participating centres over 30 months, up to 2000 children could be included."

6- A detailed description of when, how often and by whom the discharge criteria will be assessed is important as this is the primary outcome.

Response: this has been clarified at lines 238-243: "Nursing staff continuously monitor discharge criteria and register them when met. Clinical fitness for discharge is checked every four hours, starting four hours after oxygen therapy is first stopped. If oxygen therapy is not initiated, this criterion is checked every four hours starting four hours after randomization. In clinical practice, the actual discharge time may be later than when all discharge criteria are met. The reasons for any delay in discharge will be documented."

7- Good luck and I look forward to the fruits of your work!

Response: we thank the reviewer for reviewing our work.

Reviewer: 2

Comments to the Author:
After analysis, well described and aligned for collection

Response: we thank the reviewer for taking the time to review our work and their positive feedback.
